# Establishing the Molecular Diagnoses in a Cohort of 291 Patients With Predominantly Antibody Deficiency by Targeted Next-Generation Sequencing: Experience From a Monocentric Study

**DOI:** 10.3389/fimmu.2021.786516

**Published:** 2021-12-17

**Authors:** Jessica Rojas-Restrepo, Andrés Caballero-Oteyza, Katrin Huebscher, Hanna Haberstroh, Manfred Fliegauf, Baerbel Keller, Robin Kobbe, Klaus Warnatz, Stephan Ehl, Michele Proietti, Bodo Grimbacher

**Affiliations:** ^1^ Institute for Immunodeficiency, University Medical Center Freiburg, Freiburg, Germany; ^2^ Center for Chronic Immunodeficiency, University Medical Center Freiburg, Freiburg, Germany; ^3^ Faculty of Biology, University of Freiburg, Freiburg, Germany; ^4^ Resolving Infection Susceptibility (RESIST) – Cluster of Excellence 2155 to Hanover Medical School, Satellite Center Freiburg, Freiburg, Germany; ^5^ Center for Integrative Biological Signaling Studies (CIBSS), University of Freiburg, Freiburg, Germany; ^6^ Department of Rheumatology and Clinical Immunology, University Medical Center Freiburg, Freiburg, Germany; ^7^ First Department of Medicine, Division of Infectious Diseases, University Medical Center Hamburg-Eppendorf, Hamburg, Germany; ^8^ Department of Rheumatology and Immunology, Hannover Medical University, Hannover, Germany; ^9^ German Center for Infection Research (DZIF), Satellite Center Freiburg, Freiburg, Germany

**Keywords:** next-generation sequencing (NGS), targeted gene panel sequencing, hypogammaglobulinemia, common variable immunodeficiency, genetic diagnosis, predominantly antibody deficiency, primary immunodeficiency

## Abstract

Predominantly antibody deficiencies (PAD) are a heterogeneous group of disorders characterized by dysfunctional antibody production, low immunoglobulin levels in serum and impaired vaccine responses. The clinical picture is variable, ranging from mild symptoms to severe complications, which may include autoimmunity, gastrointestinal disease, allergy, and malignancies. If left untreated, PAD patients are at risk of enduring disease progression, irreversible organ damage, and reduced life expectancy. A timely diagnosis has been shown to significantly improve disease prognosis. Here, we report on our experience using targeted gene panel sequencing by employing Agilent’s HaloPlex or SureSelect and Illumina’s MiSeq technologies in a cohort of 291 individuals who presented with low or absent immunoglobulin levels in combination with or without other clinical features. In total, we have detected over 57 novel or previously reported relevant mutations in *ADA, ADA2, BTK, CTLA4, LRBA, NFKB1, NFKB2, PIK3CD, STAT3*, and *TNFRSF13B*. Overall, a genetic diagnosis could be made in 24.7% of the investigated patients. The percentage of coverage for the targeted regions ranged from 90% to 98% in this study. Moreover, functional assays were performed on a defined group of the patients carrying candidate variants in *CTLA4*, *LRBA*, *NFKB1* and *BTK*, which confirmed their deleterious effect on protein expression and/or function. This study reiterates that the immunological heterogeneity of predominantly antibody deficiencies may have a diverse genetic origin, although certain clinical features may hint towards a specific group of defects. Employing targeted sequencing panels proves to be a very time- and cost-efficient, yet reliable, method for the establishment of a genetic diagnosis in individuals with PAD. However, in case of negative panel results, or if functional testing reveals inconspicuous observations in patients with a clear indication for genetic testing, further work-up including whole exome or whole genome sequencing should be considered.

## Introduction

Predominantly antibody deficiencies (PAD) are the most common form of inborn errors of immunity (IEI); they can present at any age and have a prevalence of approximately 1:10.000 (1). PAD comprise a diverse group of immune disorders characterized by increased susceptibility to multiple, recurrent and/or severe infections, impaired antibody production and poor response to vaccines (1). Among PAD, but also in this cohort, common variable immunodeficiency (CVID) is the most clinically important form of PAD, due to its relative prevalence (1:25.000 to 1:50.000) and the number of medical encounters (2). In addition, CVID is considered as a heterogeneous and intricate disorder since some individuals present almost with complete absence of all major immunoglobulin isotypes, while others have a reduction of one, two or three immunoglobulin isotypes, in variable combinations. Non-infectious complications, such as autoimmune conditions, lymphoid hyperplasia, granulomatous inflammation, and gastrointestinal inflammatory disease, have been observed in around 30 to 50% of CVID patients (3). However, the most common genetically diagnosed form of PAD is the X-linked agammaglobulinemia (XLA), which is caused by mutations in the Bruton’s tyrosine kinase (*BTK*), and leads to a severe reduction of all serum immunoglobulin isotypes and absence of B cells (4). Additional forms of PAD may present with deficiency of one immunoglobulin isotype and with a milder clinical phenotype, as it is observed in patients with selective IgA deficiency, selective IgM deficiency or selective polysaccharide antibody deficiency (5). Up to date, about 40 different gene defects have been identified to primarily affect antibody production (6, 7); however, the genetic etiology is still unknown in up to 70-80% of patients (8). Noteworthy, the majority of CVID cases occur sporadically, and only 10 to 20% of the cases have a family history hinting towards a genetic origin. The latter is also observed in patients with selective IgA deficiency (9, 10).

The high percentage of unsolved cases might be due to limited genotype-phenotype correlations, polygenic traits, environmental factors, epigenetic causes and/or other genetic modifiers, as well as the lack of functional tests that could evaluate the deleteriousness of certain variants of uncertain significance (VUS).

In the last 10 years, the implementation of Next-Generation Sequencing (NGS) technologies have proven to be crucial in identifying the underlying genetic cause of many IEI (6, 11). Particularly, PAD-causing or PAD-associated mutations have been reported in more than 40 genes, according to the latest IEI classifications from the IUIS (6, 7). Many of these genes are not exclusively expressed in B cells, thereby leading to a more complex and variable clinical presentation in addition to hypogammaglobulinemia.

Depending on the underlying gene defect, patients might initially be diagnosed with PAD or CVID; however, as the disease progresses, additional viral and fungal infections, lung disease, autoimmune manifestations, autoinflammation, granulomatosis and/or malignancies can develop, complicate or dominate the clinical picture (12–14). Those manifestations indicate a more profound impairment and/or dysregulation of different components of the immune system. Consequently, some of the above-mentioned genetic defects are also found in patients diagnosed with combined immunodeficiency (CID) (e.g. mutations in *ICOS*, *LRBA*), with a CVID-like phenotype (e.g. *PLCG2*) (5), or with an immune dysregulation syndrome (e.g. *CTLA4*). These observations highlight the complexity of the pathomechanisms involved in PAD and CVID, since defects in B cell development, T-dependent and T-independent B cell activation, as well as in class switch recombination, have been shown to lead to hypogammaglobulinemia. These molecularly heterogeneous and clinically overlapping phenotypes challenge physicians when a solid diagnosis needs to be established. Therefore, genetic characterization in patients diagnosed with the different forms of PAD (including CVID, late-onset CID (loCID), or a CVID-like phenotype), is essential for an early molecular diagnosis. A genetic diagnosis may ensure a timely and appropriate treatment that prevents life-threatening infections and irreversible organ damage. Likewise, a molecular diagnosis helps with patient and family counselling and improves disease prognosis (1, 15).

In this study, we report our experience over the last 6 years employing targeted Next-Generation Sequencing (based on Agilent’s HaloPlex or SureSelect designs and Illumina’s MiSeq technologies) for a group of known disease-causing and other candidate genes in a cohort of 291 patients with PAD. Our purpose was to provide with a first-line genetic test to identify novel or known pathogenic variants in patients with PAD.

## Materials & Methods

### Patients

This study was conducted under the following ethics protocols: Vote no. 295/13 version 200149, Vote no. 60/18, Vote no. 290/13, and Vote no. 93/18 of the ethics committee of the University of Freiburg, Germany. All patients and their parents (when patients were under 18 years of age) were consented to participate in our study according to local ethics guidelines. Whole blood samples from 291 patients, who presented - among other features - with recurrent infections and reduced immunoglobulin levels (only one of the major isotypes: IgA, IgG or IgM, or more than one, or IgG subclass deficiency), were collected in our outpatient clinic. Patients with low levels of immunoglobulins secondary to other diseases (e.g. kidney failure, hematologic neoplasms) or secondary to pharmacologic therapies (e.g. anti-epileptic or immune-suppressive drugs) were excluded from this study. In the 291 selected individuals no previous genetic testing had been performed, 284 were sporadic cases and seven were from three unrelated multiplex families.

In contrast to previous reports on PAD patients, no participant in our study was born to consanguineous parents. Familial segregation was studied when DNA samples from parents and siblings were available.

### Panel Design

Between February 2014 and May 2020, various customized (Tier 1 or Tier 2) targeted panels were designed using Agilent’s web-based SureDesign application. All panels included genes known to cause various types of inborn errors of immunity (IEI), but optionally also included additional putative candidate genes not previously associated with disease. The first panel (ID 3, **Supplementary Table 1**) initially comprised 27 genes, and over time, our IEI panel was updated regularly in order to include novel IEI-causing genes and to optimize sequencing depth and coverage, but still fitting the probe size of Agilent’s Tier 1/2. In total 18 different panel designs were used in this study to screen the 291 individuals. Our latest and largest panel (ID 33) contained a total of 140 genes in 2020 (**Supplementary Table 1**).

### DNA Extraction, Library Preparation and Sequencing

DNA extraction from peripheral blood samples treated with EDTA was performed according to our local protocol. Briefly, erythrocytes were lysed with our in-house RBC buffer. The remaining whole peripheral leukocytes were subjected to Qiagen Cell Lysis Solution (Qiagen, Hilden, Germany) for at least 24 hours at room temperature. Qiagen protein Precipitation Solution was used to precipitate the proteins. The DNA was then precipitated with isopropanol, washed with 70% ethanol and resuspended and stored in Qiagen DNA Hydration Solution. Concentration and purity were measured by fluorometric quantification (Qubit, Invitrogen/ThermoFisher Scientific, Langenselbold, Germany). Sample preparation, target enrichment and library preparation were performed using Agilent’s HaloPlex or SureSelect enrichment system for Illumina sequencing following the manufacturer’s instructions as detailed in Agilent’s user manual (Illumina, Berlin, Germany; Agilent, Waldbronn, Germany). In brief, DNA samples were subjected to digestion by adding a restriction enzyme master mix prepared following the manufacturers protocol and an incubation step at 37°C. The digestion was validated by gel electrophoresis. Subsequently, the restriction fragments were hybridized to the HaloPlex or SureSelect probe capture library by addition of the Hybridization Master Mix as well as the indexing primer cassettes. After an incubation step, the hybridized DNA fragments were captured with a biotin-streptavidin system using HaloPlex magnetic beads. After a washing step, the circular fragments were closed through a ligation reaction, i.e. the ligation master mix was added and the solution was incubated at 55°C. Subsequently, the captured target libraries were amplified by PCR as suggested with the master mix prepared according to manufacturer’s instructions. In a final step, the amplified target libraries were purified using AMPure XP beads and washed in 70% ethanol. Enrichment was validated on an Agilent TapeStation system. Then, samples were pooled in equimolar amount for multiplex sequencing on an Illumina MiSeq system following the manufacturer’s protocol.

### Bioinformatic Analysis and Variant Interpretation

Raw sequencing data (.fast files) were pre-processed according to GATK’s best practices and included the following steps:.fastq file conversion into unmapped.bam files (PICARD tool: FastqToSam), tagging of illumine adapter sequences (PICARD tool: MarkIlluminaAdapters), conversion of tagged unmapped.bam file to.fastq file (PICARD tool: SamToFastq), sequence alignment to the human reference build hg38 (BWA MEM), identification of duplicated reads with PICARD tool: MarkDuplicates, and.bam file recalibration plus indel realignment. Variant calling was performed with GATK Haplotype caller, FreeBayes and SAMtools (16). The variants were then merged using custom BASH and R scripts, which included the unification of dinucleotide changes. Variant annotation was done using the Variant Effect Prediction (VEP) tool from ENSEMBL (https://www.ensembl.org/info/docs/tools/vep/index.html) and all results were imported into our internal database, which contains expert curated gene and variant information, and genetic (whole exome, targeted gene panel or single gene screening) and clinical data on more than 3,000 individuals. Short lists of candidate variants were generated from the database based on an (individual) frequency below 2% in our internal cohort or below 1% in the Genome Aggregation Database (gnomAD) - exomes and genomes - cohort, and a “high” or “moderate” predicted impact (**Supplementary Table 2**). However, published polymorphisms or risk alleles with a frequency up to 10% were also included. Variants were reported on the gene transcript with the highest predicted impact; however, the effect on additional gene transcripts were also available. The clinical relevance of all candidate variants was assessed following the updated guidelines (Sherloc) for the interpretation of sequence variants by the American College of Medical Genetics and Genomics–Association for Molecular Pathology (ACMG-AMP) (17). Most candidate variants were confirmed by assessing the aligned read pairs with the Integrated Genomics Viewer (IGV; Broad Institute) and, when required, validated by Sanger sequencing according to the standard protocols. In addition, familial segregation was studied when samples were available. Finally, as suggested by the guidelines, a deep literature review was performed in order to confirm whether the genetic variations found in our study were previously reported, and if gene-disease correlations and/or experimental data demonstrating a detrimental effect had already been performed.

### Variant Evaluation by Functional Assays

In order to evaluate the molecular and cellular consequence and prove the possible pathogenicity of some specific variants of interest, experimental tests measuring protein expression, phosphorylation, and/or function, were carried out in our laboratory. For this purpose, peripheral blood mononuclear cells (PBMCs) from affected patients and unaffected (travel or in-house) controls were used. In brief, PBMCs were isolated by density centrifugation and cultured with RPMI (Gibco/Thermo Fisher Scientific) medium supplemented with 10% fetal calf serum (Sigma-Aldrich/Merck, Darmstadt, Germany), 1 μg/ml penicillin and 1 μg/ml streptomycin (Invitrogen/Thermo Scientific). CTLA-4 transendocytosis and LRBA expression were assessed by flow cytometry as previously described (18–20). B-cell receptor (BCR) signaling assay (phosphorylation of Igα, SLP65 and BTK) and Ca^2+^ mobilization was determined as described before (21, 22). Data are shown after gating on naïve IgM+CD27-CD21+ or naive IgG-IgA-CD27-CD21+ B cells, respectively. Levels of Adenosine deaminase 1 or 2 (ADA and ADA2) were evaluated by measuring the specific enzyme activity in Michael S. Hershfield’s lab at Duke University School of Medicine (USA) and at the Advanced Diagnostic Unit, University of Freiburg (Germany), respectively. *NFKB1* variants were analyzed as described previously (23).

## Results

### Clinical and Genetic Characterization of 291 Patients With Suspected PAD

**Figure 1:**
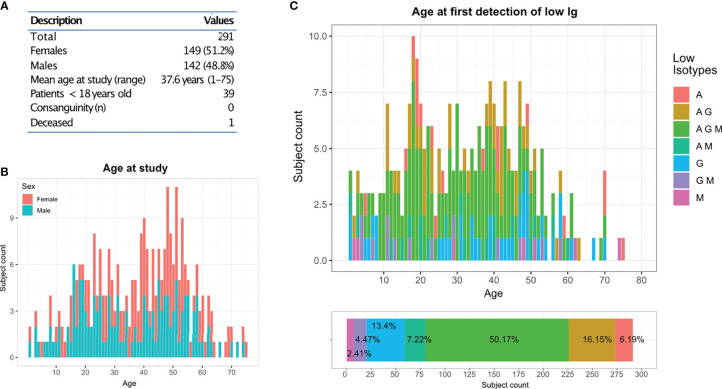
CCI Freiburg cohort: **(A)** Description of the cohort screened by targeted gene panel sequencing (TGP). **(B)** Age and gender distribution of the cohort at the time of the study. **(C)** Distribution of the cohort according to their reduced immunoglobulins profile and their age at first detection.

In this clinical and genetic study, we included a total of 291 patients who were seen at the outpatient clinic of the CCI in Freiburg and presented with hypogammaglobulinemia (reduction of at least one major immunoglobulin isotype) and a history of unusual or recurrent infections or other manifestations suggestive of altered immunity. There were 284 singleton cases and seven familial cases from three unrelated kindreds (F014: 2 sisters; F018: 3 cousins; F123: mother and daughter). Demographic features of this cohort are summarized in **Figure 1A**. The distribution age in this study was broad, with two main peaks in the second and fifth decade of life (**Figures 1A, B**). No history of consanguinity was reported. One patient deceased during the time of the study. Based on the immunoglobulin profile, reduction of all major isotypes (IgG, IgA and IgM) was reported in 50.1% of the patients, whereas 27.8% only had two out of the three isotypes reduced (IgG/IgA: 16.1%; IgM/IgA: 7.2% and IgG/IgM: 4.4%). Furthermore, 22% of the patients presented with either reduced IgG only (13.4%), IgM only (2.4%) or IgA only (6.1%). The age at first detection follows a normal distribution with two peaks at the second and fourth decade of life (**Figure 1C**).

### Genetic Characterization Informative in up to 25% of the Investigated Cases

Sequencing of all 291 patients was performed between February 2014 and May 2020 and distributed in 45 runs, employing 18 different custom targeted gene panel (TGP) designs, which were based either on Agilent’s HaloPlex or SureSelect capture methods (**Supplementary Table 1** and **Supplementary Figure 1**). Distribution of the mean reading depth per sample varied across different runs and ranged from 300x to 4,200x for samples sequenced using HaloPlex, and from 50x to 1,700x for samples sequenced with SureSelect (**Supplementary Figure 1A**). Mean base pair coverage per sample was above 90% for most samples using HaloPlex, and uniformly above 98% for samples sequenced with SureSelect (**Supplementary Figure 1B**).

As expected, the number of variants identified in each sample positively correlated with the total number of base pairs and genes sequenced (**Supplementary Figure 2**). The total number of unfiltered variants per individual ranged from 50 to 1,600 (**Supplementary Figure 2A**). The number of rare variants (frequency below 1% in internal and/or external datasets) per individual ranged from 0 to 80 (**Supplementary Figure 2B**); and the number of candidate variants (rare variants with a “high” or “moderate” predicted impact) varied between 0 to 20 (**Supplementary Figure 2C**). Because 18 different panel designs were employed to sequence all samples, and because several samples were included in more than one experiment, the number of genes screened per individual (range: 20 to 204) varied across the cohort (**Supplementary Figure 2D**).

**Figure 2:**
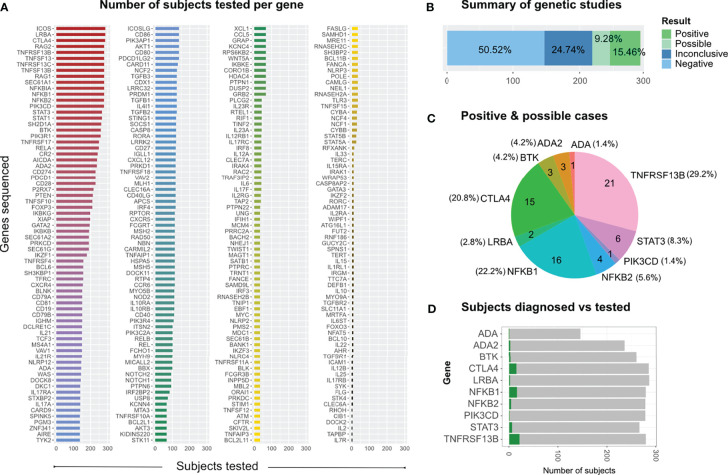
Genetic screening and findings. **(A)** All genes sequenced across individual runs from 2014 to 2020 according to the number of subjects tested per gene. **(B)** Distribution of study results, which were classified as positive (definite), possible, inconclusive and negative (no relevant variants identified). **(C)** Distribution of relevant genetic findings. **(D)** Subjects diagnosed vs subjects tested per gene, limited to those 10 genes in which we found disease relevant mutations.

In this study, we screened more than 200 genes; however, some genes were sequenced only in a few patients, while others were sequenced in more than 285 subjects (**Figure 2A**). As expected, the genes in which we found several mutations were those that had been sequenced more often (**Supplementary Figures 2A, C, D**); except for genes such as *ADA*, which had been sequenced less than 150 subjects.

In 72 of the 291 patients included in the study, we were able to identify at least one genetic variant, which we considered pathogenic or likely pathogenic (**Table 1**) following the *Sherloc* guidelines (17). In these 72 patients, we identified 57 different genetic variants, allowing us to achieve a definite molecular diagnosis in 45 patients (15.5%) and a possible molecular diagnosis in 27 patients (9.3%) (**Figures 2B–D**). This accounts for a positive hit-rate of up to 24.7%. These 57 mutations comprised 28 missense, 13 frameshift, 9 nonsense and 7 splice-site variants in the following genes: *TNFRSF13B*, *CTLA4*, *NFKB1*, *STAT3*, *BTK*, *NFKB2*, *ADA2*, *LRBA*, *ADA* and *PIK3CD* (**Table 1**). Forty-Six of those 57 relevant variants had been previously associated with disease in the literature. Furthermore, we identified 11 novel variants, which we considered likely disease-causing mutations, in 10 patients. In addition to the 57 relevant variants, 24 additional genetic variants of uncertain clinical significance (VUS) were identified in 16 of the 72 definite/possible cases (**Table 2**). In the remaining 219 patients, we only detected one or more VUS plus benign or likely benign variants, which were not sufficient to obtain a clear and conclusive molecular diagnosis and thus classified as ‘inconclusive’ (72 cases, 24.7%); or we detected benign variants or non-benign variants that did not fit with the mode of inheritance, and thereby were not considered disease-relevant and classified as ‘negative’ (147 cases, 50.5%) (**Figure 2B**).

**Table 1:** Detected mutations: Summary of detected disease-relevant variants by targeted panel sequencing (TGP).

Patient ID	Family ID	Gender (M/F)	Age at Diagnosis (years)	Molecular diagnosis		Gene	Chr. Location (GRCh38)	DNA change	Protein change	Reference sequence	Zygosity	Variant classification	Published (Variant/patient)	Reference
P002	F002	M	40.81	CHAI		*CTLA4*	2:203871449	c.531_544del	p.Phe179Cysfs*29	ENST00000648405.2	Het	Pathogenic	Subject no. 87 or MM.II.1	(24, 25)
P005	F005	F	39.0	CVID12		*NFKB1*	4:102584821	c.1066+1G>C	predicted p.Phe310Ilefs*76 (if exon 11 is skipped)	ENST00000226574.9	Het	Pathogenic	Yes	(26)
P014	F014	F	56.0	CHAI		*CTLA4*	2:203868052	c.109+1G>T	NA	ENST00000648405.2	Het	Pathogenic	Family C	(18, 24, 25)
P015	F014	F	42.87	CHAI		*CTLA4*	2:203868052	c.109+1G>T	NA	ENST00000648405.2	Het	Pathogenic	Family C	(18, 24, 25)
P017	F017	M	25.0	CHAI		*CTLA4*	2:203870883	c.407C>T	p.Pro136Leu	ENST00000648405.2	Het	Pathogenic	Subject no. 17	(24, 25)
P018	F018	M	17.0	CHAI		*CTLA4*	2:203868047	c.105C>A	p.Cys35*	ENST00000648405.2	Het	Pathogenic	Family A	(18, 24)
P020	F018	F	19.0	CHAI		*CTLA4*	2:203868047	c.105C>A	p.Cys35*	ENST00000648405.2	Het	Pathogenic	Family A	(18, 24)
P021	F018	F	28.0	CHAI		*CTLA4*	2:203868047	c.105C>A	p.Cys35*	ENST00000648405.2	Het	Pathogenic	Family A	(18, 24)
P033	F033	F	12.0	CVID2		*TNFRSF13B*	17:16948873	c.310T>C	p.Cys104Arg	ENST00000261652.6	Het	Risk allele	Yes (variant)	(9, 27, 28)
						*TNFRSF13B*	17:16940415	c.542C>A	p.Ala181Glu	ENST00000261652.6	Het	Risk allele	Yes (variant)	(29–32)
P039	F039	F	13.0	CVID12		*NFKB1*	4:102582898	c.872delA	p.Asn291Metfs*141	ENST00000226574.9	Het	Pathogenic	Yes	(26)
P048	F048	F	50.12	DADA2		*ADA2*	22:17209606	c.68_71del	p.Phe23Serfs*7	ENST00000399837.8	Hom	Pathogenic	No	–
P053	F053	M	35	CHAI		*CTLA4*	2:203870636	c.165_190dup	p.Gly64Alafs*17	ENST00000648405.2	Het	Pathogenic	No	–
P056	F056	M	40.61	CHAI		*CTLA4*	2:203870832	c.356T>G	p.Leu119Arg	ENST00000648405.2	Het	Pathogenic	Yes^§^ (variant)	(24)
P059	F059	M	55.53	CVID8		*LRBA*	4:150350017	c.7370C>G	p.Ser2457*	ENST00000357115.8	Het	Pathogenic	No	–
						*LRBA*	4:150828208	c.5143C>T	p.Gln1715*	ENST00000357115.8	Het	Pathogenic	No	–
P064	F064	M	23.70	CVID2		*TNFRSF13B*	17:16940442	c.515G>A	p.Cys172Tyr	ENST00000261652.6	Het	Risk allele	Yes (variant)	(27, 29, 30, 33)
P069	F069	F	37.28	CVID12		*NFKB1*	4:102576988	c.520_521insCTGA	p.Leu176*	ENST00000226574.9	Het	Pathogenic	Yes (variant)	(26)
P073	F073	M	21.0	CVID12		*NFKB1*	4:102578938	c.634_656dup	p.Phe220Trpfs*40	ENST00000226574.9	Het	Pathogenic	No	**-**
P093	F093	F	18.0	CVID2		*TNFRSF13B*	17:16940415	c.542C>A	p.Ala181Glu	ENST00000261652.6	Het	Risk allele	Yes (variant)	(29–32)
P098	F098	M	27.0	CHAI		*CTLA4*	2:203870699	c.223C>T	p.Arg75Trp	ENST00000648405.2	Het	Pathogenic	Subject no. 83	(18)
P101	F101	F	73.0	IMAD1		*STAT3*	17:42322384	c.1999G>T	p.Val667Leu	ENST00000264657.9	Het	Likely pathogenic	Yes^+^ (variant)	(34)
P103	F103	F	21.22	CVID2		*TNFRSF13B*	17:16940415	c.542C>A	p.Ala181Glu	ENST00000261652.6	Het	Risk allele	Yes (variant)	(29–32)
P108	F108	M	50.0	CVID12		*NFKB1*	4:102613501	c.2671delG	p.Ala891Glnfs*6	ENST00000226574.9	Het	Pathogenic	Yes (variant)	(35)
P122	F122	F	25.0	CHAI		*CTLA4*	2:203870802	c.326G>A	p.Gly109Glu	ENST00000648405.2	Het	Likely Pathogenic	Yes^§^ Subject 127	(24)
P123	F123	F	52.0	CVID10		*NFKB2*	10:102402138	c.2557C>T	p.Arg853*	ENST00000369966.8	Het	Pathogenic	Pt#22 of Fam1404	(36)
P124	F123	F	24.48	CVID10		*NFKB2*	10:102402138	c.2557C>T	p.Arg853*	ENST00000369966.8	Het	Pathogenic	Pt#23 of Fam1404	(36)
P125	F125	M	36.75	CVID2		*TNFRSF13B*	17:16948873	c.310T>C	p.Cys104Arg	ENST00000261652.6	Het	Risk allele	Yes (variant)	(9, 27, 28)
P134	F134	M	58.0	CVID12		*NFKB1*	4:102566997	c.269A>C	p.Tyr90Ser	ENST00000226574.9	Het	Likely Pathogenic	Yes (variant)	(26)
P135	F135	M	50.41	CVID2		*TNFRSF13B*	17:16940378	c.579C>A	p.Cys193*	ENST00000261652.6	Het	Risk allele	Yes (variant)	(27)
P138	F138	M	51.94	CVID2		*TNFRSF13B*	17:16948873	c.310T>C	p.Cys104Arg	ENST00000261652.6	Het	Risk allele	Yes (variant)	(9, 27, 28)
P141	F141	F	51.13	DADA2		*ADA2*	22:17209538	c.140G>C	p.Gly47Ala	ENST00000399837.8	Het	pathogenic	Patient 4	(37–39)
P143	F143	M	29	CVID10		*NFKB2*	10:102402284	c.2611C>T	p.Gln871*	ENST00000369966.8	Het	Pathogenic	Yes (variant)	(40)
P153	F153	M	59.0	CVID12		*NFKB1*	4:102576937	c.469C>T	p.Arg157*	ENST00000226574.9	Het	Pathogenic	Yes	(26)
P154	F154	M	32.0	IMAD1		*STAT3*	17:42333990	c.857A>C	p.Glu286Ala	ENST00000264657.9	Het	Pathogenic	Yes (variant)	(41)
P156	F156	F	35.0	CVID12		*NFKB1*	4:102580641	c.835+2T>G	p.Lys244_Asp279delinsAsn	ENST00000226574.9	Het	Pathogenic	Yes	(26, 42)
P160	F160	F	53.0	CVID12		*NFKB1*	4:102596201	c.1365del	p.Val456*	ENST00000226574.9	Het	Pathogenic	Yes	(26)
P163	F163	M	49.0	CVID12		*NFKB1*	4:102612609	c.2592+3A>G	Predicted p.Asp808Leufs*22 if exon 22 is skipped; retaining intron 22 predicts p.Ser866_Lys968delins10	ENST00000226574.9	Het	Pathogenic	No	–
P170	F170	M	11.58	IMAD1		*STAT3*	17:42333984	c.863A>C	p.Gln288Pro	ENST00000264657.9	Het	Pathogenic	Yes (variant)	(41)
P173	F173	F	11.0	CVID8		*LRBA*	4:150852870	c.2836_2839del	p.Glu946*	ENST00000357115.8	Het	Pathogenic	Yes (variant)	(43)
						*LRBA*	4:150908407	c.1420C>T	p.Gln474*	ENST00000357115.9	Het	Pathogenic	Yes (variant)	(43)
P182	F182	M	40.30	IMAD1		*STAT3*	17:42316899	c.2147C>T	p.Thr716Met	ENST00000264657.9	Het	Pathogenic	Yes (variant)	(44, 45)
P188	F188	M	4.32	CVID12		*NFKB1*	4:102578955	c.646A>G	p.Met216Val	ENST00000226574.9	Het	Pathogenic	Yes	(26)
P192	F192	F	53.0	CVID12		*NFKB1*	4:102580641	c.835+2T>G	p.Lys244_Asp279delinsAsn	ENST00000226574.9	Het	Pathogenic	Yes	(26, 42)
P196	F196	F	9	IMAD1		*STAT3*	17:42334008	c.839A>C	p.Gln280Pro	ENST00000264657.9	Het	Pathogenic	Yes (variant)	(41)
P198	F198	M	46.0	DADA2		*ADA2*	22:17182620	c.1223G>A	p.Cys408Tyr	ENST00000399837.8	Het	Pathogenic	Patient 2	(37)
						*ADA2*	22:17207070	c.542+1G>A	NA	ENST00000399837.8	Het	Pathogenic	Patient 2	(37)
P206	F206	M	52.0	CVID2		*TNFRSF13B*	17:16948873	c.310T>C	p.Cys104Arg	ENST00000261652.6	Het	Risk allele	Yes (variant)	(9, 27, 28)
						*TNFRSF13B*	17:16940415	c.542C>A	p.Ala181Glu	ENST00000261652.6	Het	Risk allele	Yes (variant)	(29–32)
P212	F212	M	55.0	CVID2		*TNFRSF13B*	17:16940415	c.542C>A	p.Ala181Glu	ENST00000261652.6	Het	Risk allele	Yes (variant)	(29–32)
P215	F215	M	49.89	XLA1		*BTK*	X:101354640	c.1723G>T	p.Gly575Cys	ENST00000621635.4	Hem	Likely Pathogenic	No	–
P217	F217	F	16.0	CHAI		*CTLA4*	2:203870627	c.151C>T	p.Arg51*	ENST00000648405.2	Het	Pathogenic	Subject no. 128**^§^**	(24)
P219	F219	M	23.42	CVID12		*NFKB1*	4:102578950	c.641G>A	p.Arg214Gln	ENST00000226574.9	Het	Pathogenic	Yes	(26)
P220	F220	M	9.0	CVID10		*NFKB2*	10:102402268	c.2596_2597del	p.Ser866Cysfs*19	ENST00000369966.8	Het	Pathogenic	Pt#49 (Fam846)	(26, 36)
P221	F221	F	50.25	DADA1		*ADA*	20:44621082	c.911T>G	p.Leu304Arg	ENST00000372874.9	Het	Pathogenic	Yes (variant)	(46)
						*ADA*	20:44621103	c.890C>T	p.Pro297Leu	ENST00000372874.9	Het	Pathogenic	No	–
P236	F236	M	47.0	CVID2		*TNFRSF13B*	17:16948873	c.310T>C	p.Cys104Arg	ENST00000261652.6	Het	Risk allele	Yes (variant)	(9, 27, 28)
						*TNFRSF13B*	17:16939723	c.706G>T	p.Glu236*	ENST00000261652.6	Het	Risk allele	Yes (variant)	(27)
P250	F250	M	48.43	CVID2		*TNFRSF13B*	17:16948978	c.204dup	p.Leu69Thrfs*12	ENST00000261652.6	Het	Risk allele	Yes (variant)	(9, 27, 28)
P251	F251	M	17.0	CVID2		*TNFRSF13B*	17:16940415	c.542C>A	p.Ala181Glu	ENST00000261652.6	Het	Risk allele	Yes (variant)	(29–32)
P258	F258	M	31.10	CHAI		*CTLA4*	2:203870886	c.410C>T	p.Pro137Leu	ENST00000648405.2	Het	Pathogenic	Subject no.97	(25)
P259	F259	M	15.0	CVID12		*NFKB1*	4:102584765	c.1012delT	p.Ser338Leufs*94	ENST00000226574.9	Het	Pathogenic	Yes	(26, 47)
P260	F260	M	22.72	CHAI		*CTLA4*	2:203870909	c.433_434insACGG	p.Thr147Argfs*8	ENST00000648405.2	Het	Pathogenic	No	–
P264	F264	F	23.0	IMAD1	*STAT3*		17:42346635	c.207C>A	p.Ser69Arg	ENST00000264657.9	Het	Likely Pathogenic	No	–
P265	F265	F	44.0	CVID2	*TNFRSF13B*		17:16948923	c.260T>A	p.Ile87Asn	ENST00000261652.6	Het	Risk allele	Yes (variant)	(9, 27, 30)
P271	F271	F	30.0	CVID2	*TNFRSF13B*		17:16948873	c.310T>C	p.Cys104Arg	ENST00000261652.6	Het	Risk allele	Yes (variant)	(9, 27, 28)
P274	F274	F	69.68	CVID12	*NFKB1*		4:102582898	c.872delA	p.Asn291Metfs*141	ENST00000226574.9	Het	Pathogenic	Yes (variant)	(26)
P281	F281	M	19.67	XLA-1	*BTK*		X:101375203	c.184C>T	p.Arg62Cys	ENST00000621635.4	Hem	Pathogenic	Yes (variant)	(48, 49)
P295	F295	M	40.30	XLA-1	*BTK*		X:101360688	c.757del	p.Val253Leufs*10	ENST00000621635.4	Hem	Pathogenic	Yes (variant)	(50)
P301	F301	F	53.2	CVID2	*TNFRSF13B*		17:16940415	c.542C>A	p.Ala181Glu	ENST00000261652.6	Het	Risk allele	Yes (variant)	(29–32)
P306	F306	F	58.0	CVID2	*TNFRSF13B*		17:16948873	c.310T>C	p.Cys104Arg	ENST00000261652.6	Het	Risk allele	Yes (variant)	(9, 27, 28)
P311	F311	F	34.0	CVID2	*TNFRSF13B*		17:16948873	c.310T>C	p.Cys104Arg	ENST00000261652.6	Het	Risk allele	Yes (variant)	(9, 27, 28)
P341	F341	F	49.78	CHAI	*CTLA4*		2:203870594	c.118G>A	p.Val40Met	ENST00000648405.2	Het	Likely Pathogenic	Yes^§^ (variant)	(51, 52)
P397	F397	F	17	IMD14A	*PIK3CD*		1:9726972	c.3061G>A	p.Glu1021Lys	ENST00000377346.9	Het	Pathogenic	Yes (variant)	(53–55)

Patient ID	Family ID	Gender (M/F)	Age at Diagnosis (years)	Molecular diagnosis		Gene	Chr. Location (GRCh38)	Coding change	Protein change	Transcript identifier	Zygosity	Variant classification	Published (Variant/patient)	Reference
P398	F398	M	18	CVID2	*TNFRSF13B*		17:16948873	c.310T>C	p.Cys104Arg	ENST00000261652.6	Het	Risk allele	Yes (variant)	(9, 27, 28)
P404	F404	M	56.1	CVID2	*TNFRSF13B*		17:16940415	c.542C>A	p.Ala181Glu	ENST00000261652.6	Het	Risk allele	Yes (variant)	(29–32)
P405	F405	M	29	CVID2	*TNFRSF13B*		17:16933171	c.451C>T	p.Arg151*	ENST00000579315.5	Het	Likely risk allele	No	–
P406	F406	F	60	CVID2	*TNFRSF13B*		17:16948873	c.310T>C	p.Cys104Arg	ENST00000261652.6	Het	Risk allele	Yes (variant)	(9, 27, 28)
P413	F413	M	15.8	CVID12	*NFKB1*		4:102537867	c.169C>T	p.Arg57Cys	ENST00000226574.9	Het	Pathogenic	Yes	(26)

M, Male; F, Female.

^§^variant published in patients with clinical manifestations of CTLA4 or CVID-like but not functionally tested.

^+^published as somatic mutation in a patient with peripheral T-cell lymphoma (PTCL) (34).

Hom, homozygous; het, heterozygous; hemi, hemizygous; CHAI, CTLA4 haploinsuficiency [OMIM: 616100]; CVID2, TACI deficiency [OMIM: 240500]; CVID8, LRBA deficiency [OMIM: 614700]; CVID10,NFKB2 deficiency [OMIM: 615577]; CVID12, NFKB1 haploinsufficiency [OMIM, 616576]; IMAD1, Infantile-Onset Multisystem Autoimmune Disease 1 [OMIM: 615952]; DADA1, Adenosine deaminase 1 deficiency [OMIM: 608958]; DADA2, Adenosine deaminase 2 deficiency [OMIM: 607575]; XLA1, X-linked agammaglobulinemia [OMIM: 300755]; IMD14A, Immunodeficiency 14A [OMIM: 615513].

* represents the premature stop/termination translation codon.

**Table 2:** Detected variants of uncertain significance in the 72 definite/possible cases.

Patient ID	Gene	Chr.Location (GRCh38)	Coding change	Protein change	Transcript identifier	Zygosity
P018	*F5*	1:169549811	c.1601G>A	p.Arg534Gln	ENST00000367797.8	Het
P033	*NOD2*	16:50729867	c.3019dup	p.Leu1007Profs*2	ENST00000300589.6	Het
P056	*NOD2*	16:50729867	c.3019dup	p.Leu1007Profs*2	ENST00000300589.6	Het
P064	*MYH9*	22:36285884	c.5131G>A	p.Ala1711Thr	ENST00000216181.10	Het
P101	*PIK3C2A*	11:17169343	c.399T>G	p.Phe133Leu	ENST00000265970.11	Het
	*NOD2*	16:50722629	c.2722G>C	p.Gly908Arg	ENST00000300589.6	Het
	*PIK3C2A*	11:17089793	c.5006A>G	p.Asn1669Ser	ENST00000265970.11	Het
P125	*NOD2*	16:50712175	c.2264C>T	p.Ala755Val	ENST00000300589.6	Het
	*P2RX7*	12:121162435	c.448G>A	p.Gly150Arg	ENST00000328963.10	Het
P138	*RELA*	11:65659712	c.513G>T	p.Arg171Ser	ENST00000406246.8	Het
P143	*PRKD1*	14:29927423	c.90_91ins6	p.Gly30_Ser31insAspPro	ENST00000415220.6	Het
	*NOTCH2NLC*	1:149390805	c.18_19insAGG	p.Gly6_Gly7insArg	ENST00000650865.1	Het
P154	*STAT1*	2:191009873	c.128+3A>G	–	ENST00000361099.7	Het
P182	*WAS*	X:48686945	c.724A>T	p.Ser242Cys	ENST00000376701.4	Hem
P212	*TNFRSF13C*	22:41925447	c.475C>T	p.His159Tyr	ENST00000291232.4	Het
P251	*GATA2*	3:12848131	c.1145delinsG	p.Val382Gly	ENST00000341105.7	Het
P274	*NOD2*	16:50712018	c.2107C>T	p.Arg703Cys	ENST00000300589.6	Het
P301	*IFIH1*	2:162279995	c.1641+1G>C	–	ENST00000263642.2	Het
	*CARD11*	7:2913449	c.2857G>A	p.Glu953Lys	ENST00000396946.8	Het
	*NLRP12*	19:53810880	c.779C>T	p.Thr260Met	ENST00000391773.5	Het
	*IRF3*	19:49663457	c.223A>G	p.Thr75Ala	ENST00000601291.5	Het
	*NFKB1*	4:102533876	c.150A>C	p.Gln50His	ENST00000226574.9	Het
	*NFKB1*	4:102533882	c.156A>C	p.Lys52Asn	ENST00000226574.9	Het
P311	*NOD2*	16:50712175	c.2264C>T	p.Ala755Val	ENST00000300589.6	Het
P405	*STAT3*	17:42329763	c.1123G>A	p.Val375Ile	ENST00000264657.9	Het

The majority of our 72 definite/possible patients were found to carry mutations in genes associated with autosomal dominant (AD) conditions, whereas a minority of patients carried disease-relevant mutations in genes associated with autosomal recessive (AR) or X-linked inheritance (XLR) (**Supplementary Figure 3B**). The mean age at first detection of hypogammaglobulinemia was 28.9 years for the positive/possible cases, and 32.7 years for the unsolved cases.

### Clinical Characteristics of the 72 Definite and Possible Cases

**Figure 3:**
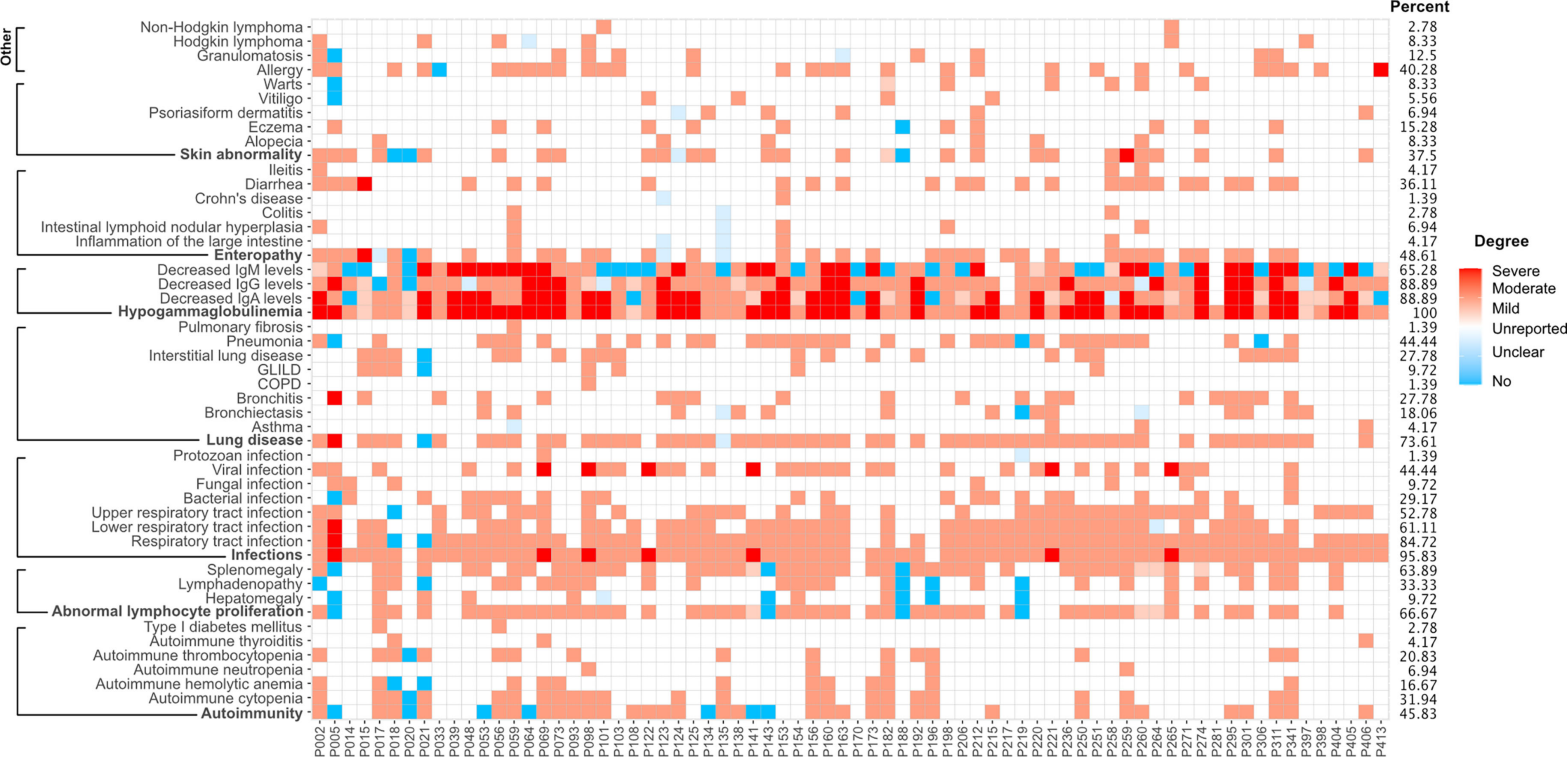
Clinical manifestations of 72 patients with a definite or possible molecular diagnosis. Presence and degree of manifestations are color-coded. Blue squares, absence; light blue squares, unclear; white squares, unreported finding; light red, mild manifestation; medium red, moderate or typical presentation; dark red squares, severe manifestation.

As mentioned above, the detection of low antibody levels - of any of the three major immunoglobulin isotypes - and a history of unusual infections (or other indication of altered immunity) were the common clinical manifestations of the entire cohort of 291 subjects. Among the 72 definite/possible cases, unusual infections (bacterial [29.2%], fungal [9.7%], viral [44.4%] or unclassified [15.3%]) were observed in 95.8% of patients. Of these, 84.7% suffered from recurrent infections of the respiratory tract, and 73.6% individuals developed lung disease, including interstitial lung disease (ILD, 37.5%), bronchiectasis (27.8%) and chronic obstructive pulmonary disease (COPD, 1.4%). Abnormal lymphocyte proliferation and gastrointestinal manifestations were found in 66.7% and 48.6% of the 72 patients, respectively. Autoimmune conditions were observed in 45.8%, and skin abnormalities (including warts) in 37.5% of individuals. Finally, 40.3% of the molecular diagnosed patients suffered from different allergies and 11.1% of patients developed lymphoma (**Figure 3**).

### Variants in *TNFRSF13B* (TACI) Are the Most Frequent Sequence Changes in the Freiburg Cohort

**Figure 4:**
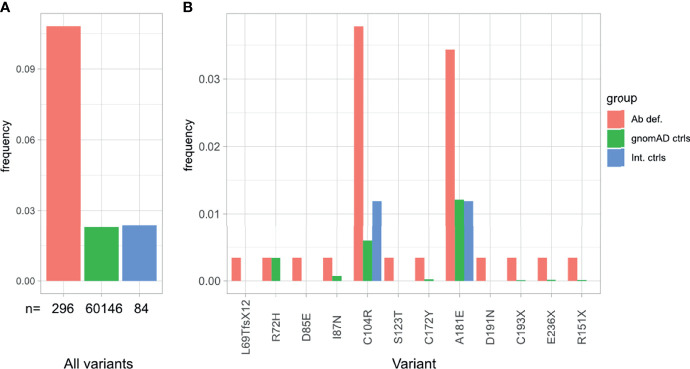
Overrepresentation of *TNFRSF13B* variants in patients with antibody deficiency compared to internal and external controls. **(A)** Frequency of *TNFRSF13B* variant carriers in our cohort in comparison to controls reported in gnomAD and internal controls from our database. **(B)** Frequency and distribution of the variants found in *TNFRSF13B* in this study, compared to their frequency in control populations.

Variants in *TNFRSF13B* were the most prevalent in our cohort, observed in 28 (9.6%) of the 291 patients in our cohort, comparing to 2.8% in controls (60,146 individuals) reported in the gnomAD database v2.1 or 2.8% in our internal control cohort (84 individuals with normal immunoglobulin levels) (**Figure 4A**). These observations are in line with previous reports on PAD or CVID cohorts (8, 56, 57).

Specifically, 12 distinct rare variants (AF < 0.01 in gnomAD exomes/genomes) in *TNFRSF13B*, were identified in 28 (9.6%) patients (**Table 3** and **Figure 4B**). Eleven out of these 12 variants were considered in this study as risk alleles and not as disease-causing since they were also found – yet at a lower frequency – in internal and external (gnomAD) controls. The remaining variant was not considered as a risk allele, since it has been reported as a polymorphism occurring at similar frequencies in affected individuals and controls (9, 58, 60). As expected, the p.Cys104Arg and p.Ala181Glu variants were found in more than 3.5% of patients, whereas the 10 additional variants were found in less than 1% of patients in this study (**Figure 4B**). Fifteen of these 28 patients carried a known monoallelic missense variant (p.Ala181Glu, p.Cys104Arg, p.Cys172Tyr, p.Ile87Asn [ENST00000261652.6]), whereas three patients carried novel monoallelic missense VUS (p.Ser123Thr, p.Asp191Asn, p.Asp85Glu). However, four patients (P002, P018, P219, P264) not only carried heterozygous missense variants in TACI (p.Arg72His, p.Ala181Glu, p.Cys104Arg), but also deleterious variants in *CTLA4* or a possibly deleterious variant in *STAT3* or *NFKB1*. Furthermore, one patient (P250) was heterozygous for a known duplication (c.204dupT) in *TNFRSF13B*, leading to a frameshift and a premature termination of translation (p.Leu69Thrfs*12), and two patients (P135 and P405) were heterozygous for the known p.Cys193* and the novel p.Arg151* (ENST00000579315.5) nonsense mutations in *TNFRSF13B*, respectively. The remaining three individuals had biallelic *TNFRSF13B* mutations: two unrelated patients (P206, P033) carried the combination of p.Cys104Arg and p.Ala181Glu, and one patient (P236) had the p.Cys104Arg mutation together with a nonsense p.Glu236* mutation. There was no noticeable difference in the clinical presentation of the heterozygous *versus* the compound heterozygous *TNFRSF13B* variant carriers. In summary, 21 of the 28 patients with variants in *TNFRSF13B* were classified as having a “possible” genetic diagnosis (**Figure 1**), whereas the three heterozygous patients with the three novel mutations were classified as “inconclusive”. The four patients with co-existing mutations in *CTLA4*, *NFKB1* and *STAT3* were classified as CTLA4, NFKB1 or STAT3 patients, respectively, but not within the group of patients carrying variants in *TNFRSF13B*.

**Table 3:** Detected mutations in *TNFRSF13B*: rare variants with an AF < 0.01 in gnomAD exomes/genomes found in 28 of 291 patients.

Patient ID	Zygosity	Chr.location	Coding change	Protein change	Variant classification	AF gnomAD controls v2.1	Published
P002	Het	17:16948968-C-T	c.215G>A	p.Arg72His	Polymorphism	0.001713	(27, 29, 58)
P013	Het	17:16948815-C-G	c.368G>C	p.Ser123Thr	Uncertain	–	No
P018	Het	17:16940415-G-T	c.542C>A	p.Ala181Glu	Risk allele	0.006136	(29–32)
P033	Het	17:16948873-A-G	c.310T>C	p.Cys104Arg	Risk allele	0.002993	(9, 27, 28)
	Het	17:16940415-G-T	c.542C>A	p.Ala181Glu	Risk allele	0.006136	(29–32)
P064	Het	17:16940442-C-T	c.515G>A	p.Cys172Tyr	Risk allele	0.000117	(27, 29, 30, 33)
P086	Het	17:16940386-C-T	c.571G>A	p.Asp191Asn	Uncertain	–	No
P093	Het	17:16940415-G-T	c.542C>A	p.Ala181Glu	Risk allele	0.006136	(29–32)
P103	Het	17:16940415-G-T	c.542C>A	p.Ala181Glu	Risk allele	0.006136	(29–32)
P125	Het	17:16948873-A-G	c.310T>C	p.Cys104Arg	Risk allele	0.002993	(9, 27, 28)
P135	Het	17:16940378-G-T	c.579C>A	p.Cys193*	Risk allele	0.000046	(27)
P138	Het	17:16948873-A-G	c.310T>C	p.Cys104Arg	Risk allele	0.002993	(9, 27, 28)
P206	Het	17:16948873-A-G	c.310T>C	p.Cys104Arg	Risk allele	0.002993	(9, 27, 28)
	Het	17:16940415-G-T	c.542C>A	p.Ala181Glu	Risk allele	0.006136	(29–32)
P212	Het	17:16940415-G-T	c.542C>A	p.Ala181Glu	Risk allele	0.006136	(29–32)
P219	Het	17:16948873-A-G	c.310T>C	p.Cys104Arg	Risk allele	0.002993	(9, 27, 28)
P236	Het	17:16948873-A-G	c.310T>C	p.Cys104Arg	Risk allele	0.002993	(9, 27, 28)
	Het	17:16939723-C-A	c.706G>T	p.Glu236*	Risk allele	0.000075	(27)
P250	Het	17:16948978-G-GT	c.204dup	p.Leu69Thrfs*12	Risk allele	0.000233	(9, 27, 28)
P251	Het	17:16940415-G-T	c.542C>A	p.Ala181Glu	Risk allele	0.006136	(29–32)
P264	Het	17:16940415-G-T	c.542C>A	p.Ala181Glu	Risk allele	0.006136	(29–32)
P265	Het	17:16948923-A-T	c.260T>A	p.Ile87Asn	Risk allele	0.000366	(9, 27, 30, 59)
P271	Het	17:16948873-A-G	c.310T>C	p.Cys104Arg	Risk allele	0.002993	(9, 27, 28)
P301	Het	17:16940415-G-T	c.542C>A	p.Ala181Glu	Risk allele	0.006136	(29–32)
P306	Het	17:16948873-A-G	c.310T>C	p.Cys104Arg	Risk allele	0.002993	(9, 27, 28)
P311	Het	17:16948873-A-G	c.310T>C	p.Cys104Arg	Risk allele	0.002993	(9, 27, 28)
P316	Het	17:16948928-G-C	c.255C>G	p.Asp85Glu	Uncertain	–	No
P398	Het	17:16948873-A-G	c.310T>C	p.Cys104Arg	Risk allele	0.002993	(9, 27, 28)
P404	Het	17:16940415-G-T	c.542C>A	p.Ala181Glu	Risk allele	0.006136	(29–32)
P405	Het	17:16933171-G-A	c.451C>T	p.Arg151*	Likely risk allele	–	No
P406	Het	17:16948873-A-G	c.310T>C	p.Cys104Arg	Risk allele	0.002993	(9, 27, 28)

* represents the premature stop/termination translation codon.

### Mutations in *NFKB1* and *NFKB2* Collectively Account for 27.8% of the Solved Cases in Our Cohort

We found that the clinical phenotype of many of our patients could be genetically explained by monoallelic *NFKB1* mutations and, less commonly, by *NFKB2* mutations (**Figure 2C** and **Table 1**). A total of 16 patients carried relevant mutations in *NFKB1*. Fourteen of whom were found to have severe N-terminal truncating mutations. These N-terminal truncating mutations lead to haploinsufficiency of both, the p105 precursor protein (encoded by *NFKB1*) and the mature p50 (which is generated by proteasome-mediated removal of the C-terminal half of p105). Patient P153 had the known stop-gain mutation p.Arg157* (61, 62). Patients P039 and P274 (from unrelated families) both carried the single base pair deletion (c.872delA; p.Asn291Metfs*141). Patient P259 had the c.1012delT; p.Ser338Leufs*94 mutation, and P069 carried a 4-base pair insertion (c.520_521insCTGA; p.Leu176*). All these individuals were reported in 2020 as part of the cohort studied by Lorenzini and colleagues (26). Patient P073 had not been previously reported and had a novel 23bp duplication (c.634_656dup; p.Phe220Trpfs*40). This mutation is also predicted to disrupt both, the precursor p105 and the mature form p50 of NF-κB1. In analogy to other well-known severely truncating mutations (63), we consider this newly identified variant as pathogenic, although we have not explicitly confirmed its deleterious effect. Patients P005, P156 and P192 (all unrelated) carried splice-altering mutations: The splice-donor change c.1066+1G>C (which was found in P005) results in a shift of the reading frame and a premature termination of translation (p.Phe310Ilefs*76); however, other splice defects are also conceivable. The change c.835+2T>G, which is found in P156 and P192, leads to in-frame skipping of exon 9 and causes an internal deletion of 36 amino acids and insertion of an asparagine residue due to the fusion of exon 8 and 10 (p.LysK244_Asp279delinsAsn) (42). These patients were also included in the Lorenzini et al. cohort (26, 42).

In addition, we identified four patients carrying four missense variants: P134 (c.269A>C; p.Tyr90Ser), P188 (c.646A>G; p.Met216Val), P413 (c.169C>T; p.Arg57Cys), and patient P219 (c.641G>A; p.Arg214Gln). The latter patient additionally carried the known p.Cys104Arg mutation in *TNFRSF13B*. Although functional *in vitro* testing, as previously described (23), indicated that none of these four variants cause a detrimental protein loss, we could not exclude a hypomorphic reduction of protein function (data not shown). Using reporter assays, a recent study demonstrated a loss-of-function for p.Arg57Cys, whereas a functional defect associated with p.Tyr90Ser, p.Arg214Gln, and p.Met216Val, remained obscure (63). P134 presented with splenomegaly, pneumonias and psoriatic dermatitis. P219 suffered from recurrent respiratory tract infections and gastrointestinal manifestations. P413 presented with severe allergy. Unfortunately, detailed clinical data from patient P188 were not available (**Figure 3**). Furthermore, patient P160, who was also included in the NFKB1 cohort reported previously (26), carried a single-nucleotide deletion (c.1365delT; p.Val456*) in the central part of *NFKB1.* This particular mutation predicts skipping the precursor p105 stage and the immediate expression of p50-like mutant proteins (64, 65). P160 presented with recurrent viral and bacterial infections, autoimmune hemolytic anemia (AIHA), abnormal lymphoproliferation, and allergy. Finally, we detected two subjects carrying truncating mutations that affect the C-terminal portion of *NFKB1*: Patient P108 harbors a single-nucleotide deletion (c.2671delG; p.Ala891Glnfs*6), which is predicted to alter the amino acid sequence of the death domain (DD) of the p105 precursor (35). Patient P163 was found to have a putative splice donor mutation (c.2592+3A>G), predicting the expression of an abnormal precursor protein (p.Asp808Leufs*22 if the variant leads to skipping of exon 22; or p.Ser866_Lys968delins10 if intron 22 is retained). To date, the specific defects of the C-terminally truncated p105 proteins remain unknown.

With regard to *NFKB2*, we identified four patients carrying disease-relevant mutations: P123 and P124 (mother and her daughter), who were previously described by Klemann et al. in 2019 as Pt#22 and Pt#23 of Fam1404 (36), carry the most-frequent dominant-negative nonsense mutation (c.2557C>T; p.Arg853*) in *NFKB2* (66, 67). Patient P143 was heterozygous for the previously published stop-gain mutation: c.2611C>T; p.Gln871* (36, 40) and suffered from recurrent upper and lower respiratory tract infections, alopecia, psoriasiform dermatitis, vitamin D deficiency, and osteoporosis. Subject P220, who was also reported in the above study as Pt#49 (Fam846) (36), carries a *de novo* heterozygous deletion (c.2596_2597delAG; p.Ser866Cysfs*19) (**Table 1**).

### Fifteen of the 291 Investigated Patients Have Mutations in *CTLA4*

**Figure 5:**
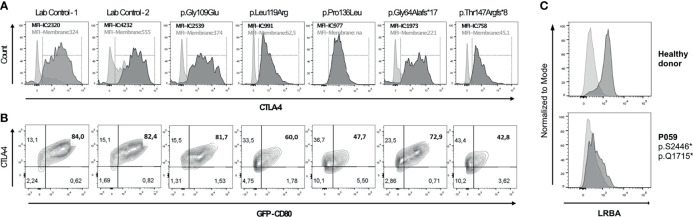
Functional assessment of novel genetic variants in *CTLA4* and LRBA individuals by flow cytometry. **(A)** Histogram overlays show CTLA-4 expression on the surface (light grey) and total intracellular CTLA-4 (dark grey) in activated CD4+ FOXP3+ (Treg) cells. **(B)** Ligand binding uptake of GFP-CD80 by stimulated primary CD4+FOXP3 primary cells of two controls and four patients. Flow cytometry plots depict the percentage of GFP-CD80. **(C)** Histogram overlays show isotype (light grey) and LRBA expression (dark grey) in peripheral blood mononuclear cells stimulated with phytohaemagglutinin (PHA) for 72h in a healthy donor (HD) and in patient 059 (P059).

Among the 291 investigated patients, 15 patients from 12 unrelated families were found to carry relevant mutations in *CTLA4*. The mutations in *CTLA4* included one single-nucleotide splice-site mutation (c.109+1G>T), which is known to affect the mRNA splicing (18). This mutation was found in two affected sisters (patients P014 and P015 from family F014). These women were initially reported in 2014 by Schubert et al. as Family C (18). Three individuals were found to carry known stop-gain mutations in residues located in the ligand-binding domain of the protein: Patient P018 from family F018, who also carried the known variant p.Ala181Glu in *TNFRSF13B*, had the p.Cys35* nonsense variant in *CTLA4* (18). This mutation is also present in two of his cousins (P020, P021). This family had also been reported before (Family A) by Schubert et al. in 2014 (18). Patient P217 had the p.Arg51* mutation (she was previously reported as subject no. 128 by Schwab et al. in 2018) (24). Three individuals were found to carry frameshift mutations: Patient P002, who was previously reported as subject no. 87 (24) or MM.II.1 (25), harbored a 14-base-pair deletion (c.530_543del; p.Phe179Cysfs*29). Patients P260 and P053 carried a novel 4-base pair insertion (c.433_434insACGG; p.Thr147Argfs*8), and a novel 35-base pair duplication (c.165_190dup; p.Gly64Alafs*17, respectively. Functional evaluation showed low levels of intracellular CTLA-4 expression and a reduced percentage of CD80-ligand uptake for the p.Thr147Argfs*8 and the p.Gly64Alafs*17 mutants (**Figure 5**). P260 and P053 suffered from recurrent and severe respiratory tract infections, which ultimately led to the development of bronchiectasis. However, P260 had additional clinical manifestations such as enteropathy, nodular lymphoid hyperplasia, ileitis, and skin abnormalities. Six patients were found to bear missense mutations, four of whom had been previously reported: P098 [subject no. 83 (24)] [p.Arg75Trp], P258 [subject no. 97 (24)] [p.Pro137Leu], P017 [subject no. 17 (24)] [p.Pro136Leu], and P122 [subject 127 (24)] [p.Gly109Glu]. Two of them, however, have previously not been reported: P341 [p.Val40Met] and P056 [p.Leu119Arg]. P341 presented with autoimmune cytopenia, recurrent lower respiratory tract infections, lymphoproliferative features and enteropathy. Patient P056 presented with type I diabetes, autoimmune thrombocytopenia (ITP), lymphadenopathy, ILD, eczema, and Hodgkin lymphoma. Nevertheless, four out of these seven missense variants had not been experimentally confirmed to functionally affect the biology of CTLA-4. Further work-up revealed reduced intracellular CTLA-4 expression for patients’ cells carrying the variants p.Pro136Leu (68) and p.Leu119Arg; whereas intracellular CTLA-4 expression in patient’s cells carrying the p.Gly109Glu variant was not affected (**Figure 5A**). Additionally, the percentage of CD80-binding ligand uptake was found to be reduced for cells carrying the variants p.Pro136Leu and p.Leu119Arg, which is consistent with the phenotype observed for other loss-of-function mutations; however, it was not reduced in the patient’s Tregs carrying the p.Gly109Glu variant (**Figure 5B**). In these cells, the percentage of transendocytosis was comparable to that observed in healthy donors (**Figure 5B**). We conclude that the functional tests conducted by us were not suitable for showing the CTLA-4 impairment caused by the p.Gly109Glu missense mutation. Patient’s PBMCs carrying the variant p.Val40Met were not available for functional testing.

### Six of the 291 Investigated Patients Carry Mutations in *STAT3*

Monoallelic gain-of-function (GOF) mutations in *STAT3* were identified in six unrelated patients. Patients P154 (c.857A>C; p.Glu286Ala) and P170 (c.863A>C; p.Gln288Pro) carried mutations that were recently shown to have an increased DNA binding affinity and baseline activity in comparison to the wild-type STAT3 (41), whereas the mutation identified in P196 (c.839A>C; p.Gln280Pro) only caused a slightly increased basal transcriptional activity, which was strongly increased after stimulation. However, the extent and duration of phosphorylation, as well as the distribution of pSTAT3 within the cell, was comparable to the wild-type STAT3 levels (41). Patient P182 carried a known GOF mutation (c.2147C>T; p.Thr716Met), which has been previously identified in patients with enteropathy and autoimmune cytopenias (44, 45). Similarly, patients P182 and P196 presented with either hepatomegaly or splenomegaly and autoimmune cytopenia. P182 and P154 had low levels of IgA and IgG2 in serum and suffered from recurrent and severe respiratory tract infections. P182 presented with vitiligo, warts and mastoiditis; whereas P154 developed GLILD, diabetes, a hematological neoplasm and suffered from recurrent herpes. P170 had thrombocytopenia, but unfortunately additional clinical information was not available. The clinical manifestations exhibited in patients P154, P182 and P196 were compatible with the clinical phenotype observed in patients with Infantile-Onset Multisystem Autoimmune Disease 1 (IMAD1) [OMIM #615952] caused by GOF mutations in *STAT3*.

Moreover, we detected two previously unreported germline missense variants in *STAT3*. P101 harbors the c.1999G>T; p.Val667Leu mutation, which has only been reported as a somatic mutation associated with the development of a T-cell lymphoma in one patient (34). Patient P264, who also carried the known p.Ala181Glu variant in *TNFRSF13B*, was found to carry an undescribed variant (c.207C>A; p.Ser69Arg) in *STAT3*. Patient P101 had a history of decreased IgA levels in serum, autoimmune cytopenia, splenomegaly, enteropathy, ILD and recurrent pneumonias; whereas patient P264 suffered from recurrent respiratory infections, atopic dermatitis, mild lymphoproliferation, celiac disease, chronic diarrhea, and arthralgias. Furthermore, P264 presented with reduced levels of all immunoglobulin isotypes. T cells from P101 and P264 showed only a slight reduction in STAT3 phosphorylation compared to controls (data not shown); therefore, these two cases were classified as possibly solved.

### Deleterious Biallelic *LRBA* Mutations in Two of the 291 Patients

We identified biallelic disease-causing mutations in two unrelated patients, who presented with very low B cell number, enteropathy, pulmonary disease and autoimmune features. LRBA is implicated in the regulation of CTLA-4 and cell survival as well as in endosomal trafficking (69–71). The four deleterious mutations identified include two novel nonsense variants: c.7370C>G; p.Ser2457* and c.5143C>T; p.Gln1715* in patient P059, and the known mutations: c.1420C>T; p.Gln474* (43) and c.2836_2839delTTTC; p.Glu946* in patient P173, who was already reported as Patient 1 (105–1) by Gámez et al. in 2016 (43). Further work-up by using flow cytometry showed severely reduced surface expression of LRBA in PBMCs from patient P059 in comparison to the healthy donor, thus suggesting that both alleles fail to produce any functional LRBA protein (**Figure 5C**).

### Four Patients With *ADA* or *ADA2* (*CECR1*) Mutations Were Identified in our Cohort

Four patients were found to carry relevant mutations either in *ADA* or in *ADA2*. Patient P221 presented with severe B lymphopenia with slightly reduced T-cell counts and hypogammaglobulinemia, suffered from recurrent respiratory infections leading to pneumonias, bronchiectasis, asthma and ILD. Furthermore, she suffered severe bacterial and viral infections including meningococcal meningitis. She was found to carry compound heterozygous mutations in *ADA* comprising one published amorphic missense variant (c.911T>G; p.Leu304Arg) and one unreported variant (c.890C>T; p.Pro297Leu). Levels of ADA enzymatic activity were undetectable with a definite increase in intracellular metabolites, thus confirming the suspected pathogenicity of both variants (data not shown).

Moreover, we identified three unrelated patients carrying biallelic mutations in *ADA2*. Patient P048 - who suffered from recurrent infections, pancytopenia, livedo reticularis and hypoalbuminemia - was found to carry a novel homozygous deletion resulting in a frameshift mutation and premature stop codon (c.68_71delAAGA; p.Phe23Serfs*7). Further work-up confirmed low levels of ADA2 enzymatic activity (data not shown). Patient P198, who was previously described as Patient 2 by Schepp et al. in 2017 (37), carried the missense change p.Cys408Tyr and the splice-site variant c.542+1G>A in compound heterozygosity. Patient P141, previously described as Patient 4 by Schepp et al. (37), carried the homozygous missense mutation p.Gly47Ala (38, 39).

### Hemizygous *BTK* Mutations in Three Male Patients

Three unrelated male patients were identified carrying hemizygous mutations in *BTK* (Bruton Tyrosine Kinase), encoding an essential kinase for development and maturation of B cells to antibody-secreting cells (72). The mutations in *BTK* included a previously described single-nucleotide deletion (c.757delC) (48) in patient P295 leading to a frameshift and premature termination (p.Val253Leufs*10) and two missense mutations: p.Arg62Cys and p.Gly575Cys, which were identified in patients P281 and P215, respectively. The variant p.Gly575Cys, to our knowledge, has not been previously reported. Further work-up to test the deleterious potential of the p.Gly575Cys mutation showed detectable BTK protein expression (**Supplementary Figure 4A**) but reduced Ca^2+^ flux in naive CD19+CD21+ B cells (**Supplementary Figure 4B**), despite normal phosphorylation of Igα, SLP65 and BTK itself (**Supplementary Figure 4C**). Ca^2+^ signaling is downstream of BTK phosphorylation suggesting that - despite normal BTK phosphorylation on Y551 - the signal transduction downstream of BTK seems to be affected in cells harboring this mutation.

### Mutation Identified in *PIK3CD*

Patient P397 was found to carry the most commonly reported GOF mutation (c.3061G>A; p.Glu1021Lys) in *PIK3CD* (53–55). She presented with recurrent infections, lung disease, bronchiectasis and Hodgkin lymphoma, consistent with the clinical phenotype observed in patients with activated PI3K delta syndrome (APDS).

## Discussion

Early clinical and molecular diagnosis of patients with PAD could avoid suffering from repeated or chronic infections and subsequent organ damage. The heterogeneous underlying genetic etiology of PADs - and IEIs in general - and the interpretation of rare or novel variants with an atypical immune phenotype further challenge the establishment of a definitive diagnosis. Particularly, VUS are disappointing for both physicians and patients, when relying on genetic testing to confirm a suspected diagnosis.

In this study we summarize our findings using NGS and a targeted gene panel (TGP) approach to analyze the genetic background of a diverse cohort of 291 individuals who presented with selective or complete antibody deficiency. The use of NGS technology coupled with the results obtained from subsequent *in vitro* functional testing allowed us to evaluate 57 possibly relevant mutations and establish a possible or definite molecular diagnosis in 72 of the evaluated patients.

If we consider all possible and definite cases, the diagnostic yield for this cohort goes up to 24.74%, which is within the range of what other studies have reported (10 to 70%) on different cohorts of IEI patients employing various NGS approaches (**Supplementary Table 3**) (8, 11, 57, 73–77). The rates of positive hits between studies varies greatly based on the method used (WES or TGP), patient pre-selection and population, percentage of consanguinity, the number of selected genes, and filtering strategies. For example, in studies including pediatric patients with an early disease onset or patients born to consanguineous parents with a marked phenotype, the likelihood of identifying the underlying genetic defect - regardless of the sequencing approach - is higher than in studies including adult patients, patients from non-consanguineous families, or patients with a less clear phenotype.

Most of the genetic studies in PAD cohorts employing a TGP or NGS approach published thus far included a variable number of IEI-related genes, ranging from 17 to 623 (**Supplementary Table 3**). In our study, we analyzed up to 287 genes known to be essential for B cell development, differentiation and activation, as well as genes important for T cell function and genes involved in other critical signaling pathways of the immune system. We observed that most of the genetically diagnosed patients carry mutations in *TNFRSF13B, NFKB1 or CTLA4*, which collectively account for 72.2% in our cohort (**Figure 2C**). The identification of disease-relevant mutations in only 10 of the 287 studied genes may be biased, since not all genes were sequenced in an equal number of patients (**Figure 2A**); however, there were 20 genes that were screened in more than 250 patients. On the other hand, even though some genes, such as *ICOS or SEC61A1*, were screened at least in 280 patients, we did not find disease-relevant variants. It is however not surprising that screening of genes such as *LRBA* or *RAG2* (also screened in more than 280 individuals) only led to the diagnosis of two individuals, since these genes are found more frequently mutated in pediatric cohorts or in individuals born from consanguineous families (which are both under-represented in our cohort). Interestingly, we found one patient with relevant mutations in *ADA*, which are also more commonly identified in pediatric cohorts. *ADA* was only screened in half of the cohort (146 patients), which suggests that if this gene had been sequenced in the entire cohort, we might have detected additional mutations in our adult cohort. However, this could be true for other genes as well.

There are technical factors that certainly influence the diagnostic yield of NGS, such as the coverage of the target regions and the sequence reading depths. Low coverage increases the likelihood of missing possibly relevant mutations. The average coverage per run in this study was above 90% for almost all regions of interest, and reached 98% when using SureSelect designs (**Supplementary Figure 1B**). In our hands therefore, SureSelect performed better than HaloPlex regarding not only the total percentage of bases covered, but also the variability between samples of the same run, or between different runs (**Supplementary Figure 1B**). Low sequence reading depths can be a limiting factor in WGS or WES; however, in our study, this was not an issue as the mean reading depth per run was about 1000x, and the run with the lowest mean reading depth had 116x (**Supplementary Figure 1A**). We also observed that the use of SureSelect designs led to a more uniform distribution of sequencing reads between samples of the same run than the use of HaloPlex designs (**Supplementary Figure 1A**). Despite the limiting factor of pre-selected genes in TGP, the superior sequencing metrics that can be achieved using this technology compared to WES or WGS makes it a reliable, cost-effective and rapid first-line approach to diagnose patients with more typical phenotypes. On the other hand, the advantage of using WES or WGS instead of TGP can enable the genetic diagnosis of patients with pathogenic variants in less common or unexpected genes. The 219 individuals for whom we did not achieve a clear molecular diagnosis, despite having a good coverage for a broad number of PAD-associated genes, should therefore be subjected to WES/WGS to investigate whether they carry disease-causing variants in other immune-related genes not included in our TGP designs or in non-coding regions. Our preliminary findings using WES in an overlapping cohort show and increased 10-15% diagnostic yield compared to the use of TGP (unpublished data), which is a yield comparable to what others have observed for different singleton and trio cohorts (56, 78–80). A follow up WES study to examine many of the unsolved cases is currently in progress. Moreover, in patients with complex diseases (e.g. CVID) it is critical to consider that two or more subtle defects present in different genes might cause the phenotype. There is accumulating evidence that at least a subgroup of CVID patients likely have an oligogenic or polygenic origin rather than a monogenic cause (81). Our results confirm previous observations that an accurate genetic diagnosis cannot be made in about 70-80% of patients with PAD using only a TGP - reflecting the broad and complex clinical spectrum of these group of patients - and that further analysis, including WES, WGS, SNP-arrays or long-read sequencing are required to increase the diagnostic yield. Furthermore, gene-specific functional assays must be available, suitable and sensitive enough to confirm or reject the pathogenicity of a particular VUS.

Autosomal recessive (AR) disorders remain four times more common than autosomal dominant (AD) disorders among described IEI (6). Nonetheless, in this study most patients (63/72) were found to carry a disease-relevant mutation in a gene that follows an AD mode of inheritance (mutations in *TNFRSF13B*, *CTLA4*, *NFKB1, NFKB2, STAT3* and *PIK3CD* genes). Six patients were found to carry compound heterozygous mutations in genes following an AR pattern of inheritance and three males were found to have hemizygous mutations in genes associated with X-linked recessive disorders. This trend in the increase of AD defects has been observed before in cohorts of CVID patients from Western countries with non-consanguineous backgrounds in the last years (8, 11).

In line with previous reports in CVID, more than 29.2% of our positive or possible cases (**Figure 2C**) were found to carry known deleterious changes in *TNFRSF13B* (11, 57, 73), although mutations in this gene are currently considered risk alleles rather than disease-causing variants, as they are also found in asymptomatic carriers (11, 82). Most of the candidate variants in *TNFRSF13B* found in this study (**Table 3**) have been repeatedly associated with some degree of antibody deficiency, compromised B cell function, higher risk of developing autoantibody-mediated autoimmunity and/or lymphoproliferation (9, 58, 83). In our cohort, variants in *TNFRSF13B* have been observed in 9.6% of patients. However, the exact same *TNFRSF13B* variants are present in approx. 2.8% of the healthy population. This points towards *TNFRSF13B* as a considerable risk gene for antibody deficiency and autoimmunity by the factor of 3.4x. Although the penetrance of this risk alleles in the general population seems to be rather small (0.0133%), the risk is approximately 35% within families with at least one PAD patient (46 of 133 *TNFRSF13B* mutation carriers from 34 multiplex families were affected by dysgammaglobulinemia; B. Grimbacher, unpublished data). This observation points either to an involvement of a second confounding genetic factor, or an environmental trigger at work in these affected families, but not in the many families with *TNFRSF13B* variant carriers without antibody deficiency. For the purpose of comparability to other publications in the field, we have decided to call the *TNFRSF13B* variants with a biological impact on TACI signaling (9) pathogenic.

Of note, we detected four patients (P002, P018, P219 and P264) each carrying known variants in *TNFRSF13B* and a second potentially pathogenic mutation in *CTLA4*, *STAT3* or *NFKB1*. We currently do not know whether the identified variants in *TNFRSF13B* (TACI) might influence or affect disease presentation and severity in patients with additional pathogenic mutations in genes associated with other well-defined IEI disorders. Due to the complexity of TACI-mediated signaling, more specific functional analysis in patients with multiple mutations is needed in order to determine the contribution of TACI variants to the overall phenotype. It is in fact conceivable that two (or more) “weakly hypomorphic” variants affecting the same signaling pathway at different steps might act synergistically to trigger a pathogenic mechanism, as some studies in CVID patients have begun to demonstrate (84).

Notably, 16 patients of the 72 possible/positive cases had candidate variants in two or more genes (**Table 2**). However, the significance of these variants in the pathogenesis of PADs is still undetermined and further experimental evidence is needed to clarify whether the presence of these additional variants may influence the course and severity of the disease. Until now, only a few genetic studies have reported on the VUS identified in their IEI patients. Yet we believe that additional variants of uncertain significance in critical genes should also be reported, particularly in patients with broad, complex or variable phenotypes, such as CVID, which might not be explainable by monogenetic defects.

Hypomorphic mutations in other IEI genes (*BTK, GATA2, IL2RG*, *JAK3*, *RAG1*, *RAG2*, etc.) have been previously found in patients with antibody deficiency and milder phenotypes or in CVID patients (85–90). Interestingly, P215, who presented with hypogammaglobulinemia, low circulating B cells, and impaired vaccine responses, carried a new hypomorphic variant in the kinase domain of BTK. Additional functional evaluations revealed mild defects in B cell activation but not in protein expression, suggesting residual function of BTK. Similar to our findings, patients with a late-onset of BTK insufficiency and less severe phenotype due to hypomorphic mutations in this gene have also been reported (88, 91). This case demonstrates the importance of also considering hypomorphic mutations in adult patients besides complete loss-of-function (amorphic) mutations.

In summary, this work highlights the need for careful evaluation of PAD patients, in order to provide a reliable molecular diagnosis and to initiate the most appropriate treatment. This evaluation should combine the clinical data and laboratory parameters with the genetic findings and functional proof from experimental assays in order to establish solid genotype-phenotype correlations and thereby reduce the number of VUS. We conclude that at least the following genes: *ADA*, *ADA2, BTK, CTLA4, LRBA, NFKB1, NFKB2, PIK3CD, STAT3* and *TNFRSF13B* should always be considered in any custom panel design intended to be used as a diagnostic test for patients with complete (reduction of all major immunoglobulin isotypes) or selective antibody deficiency.

## Data Availability Statement

The datasets presented in this article are not readily available because uploading the data is not part of the participants consent according to Art. 7 GDPR. Requests to access the datasets should be directed to michele.proietti@uniklinik-freiburg.de or bodo.grimbacher@uniklinik-freiburg.de.

## Ethics Statement

The studies involving human participants were reviewed and approved by the Ethics committee of the University of Freiburg, Germany. Written informed consent to participate in this study was provided by the participants’ legal guardian/next of kin.

## Author Contributions

JR-R and AC-O conceived, analyzed, interpreted the data and wrote the manuscript. JR-R, KH, HH, MF, and BK performed experiments and analyzed the results. AC-O and MP developed the internal database and bionformatics analysis pipeline. RK, KW, SE, and BG provided patient care, collected and provided clinical data, and commented on the manuscript. BG and MP designed and supervised the project, provided resources and edited the manuscript. All co-authors reviewed, commented and approved the final version of the manuscript.

## Funding 

This work was funded by the Deutsche Forschungsgemeinschaft (DFG) SFB1160/2_B5, under Germany's Excellence Strategy (CIBSS – EXC-2189 – Project ID 390939984, and RESIST – EXC 2155 – Project ID 390874280); by the E-rare program of the EU, managed by the DFG, grant code GR1617/14-1/iPAD; and by the German Federal Ministry of Education and Research (BMBF) through a grant to the German Auto-Immunity Network (GAIN), grant code 01GM1910A. This work was supported in part by the Center for Chronic Immunodeficiency (CCI), Freiburg Center for Rare Diseases (FZSE). Some samples have been taken from the CCI-biobank, a partner of the Freeze Biobank Freiburg. Flow cytometry and cell sorting was performed at the Lighthouse Core Facility of the Medical Faculty, Freiburg.

## Conflict of Interest

The authors declare that the research was conducted in the absence of any commercial or financial relationships that could be construed as a potential conflict of interest.

## Publisher’s Note

All claims expressed in this article are solely those of the authors and do not necessarily represent those of their affiliated organizations, or those of the publisher, the editors and the reviewers. Any product that may be evaluated in this article, or claim that may be made by its manufacturer, is not guaranteed or endorsed by the publisher.
