# Supplementary material for: Establishing the Molecular Diagnoses in a Cohort of 291 Patients With Predominantly Antibody Deficiency by Targeted Next-Generation Sequencing: Experience From a Monocentric Study
Source: Front Immunol. 2021 Dec 17;12:786516. doi: 10.3389/fimmu.2021.786516 (PMC8718408; doi:10.3389/fimmu.2021.786516)
Supplement: Supplementary file 1 [file DataSheet_1.pdf]

ESTABLISHING THE MOLECULAR DIAGNOSES IN A COHORT OF 291 PATIENTS WITH PRIMARY ANTIBODY DEFICIENCY BY TARGETED NEXT-GENERATION SEQUENCING: EXPERIENCE FROM A MONOCENTRIC STUDY.

- SUPPLEMENTARY MATERIAL -

Supplementary Table 1. Genes sequenced per panel (18 in total) from 2014 to 2020.

Panel ID	Gene list
3	BCL6, CTLA4, GATA2, ICOS, IKBKB, IKBKG, LRBA, NFKB1, NFKB2, NFKBIA, PIK3CD, PRKCD, RAG1, RAG2, RELA, RIF1, SEC61A1, SEC61A2, SEC61G, SH3KBP1, TFRC, TNFRSF13B, TNFRSF13C, TNFRSF17, TNFRSF4, TNFSF13, TNFSF13B
5	AICDA, BLNK, BTK, CD19, CD27, CD40, CD40LG, CD79A, CD79B, CD81, CR2, FCGR2B, ICOS, IGHM, IGLL1, IKBKB, IKBKG, LRBA, NFKB1, NFKBIA, PTPN22, RAG1, RAG2, RIF1, SEC61A1, TNFRSF13B, TNFRSF13C, TNFRSF17, TNFRSF4, TNFSF13, TNFSF13B
6	AKT1, BCL6, BTK, CD274, CD28, CD80, CD86, CTLA4, GATA2, ICOS, ICOSLG, IKBKB, IKBKG, LRBA, NFKB1, NFKB2, NFKBIA, PDCD1, PDCD1LG2, PIK3AP1, PIK3CD, PIK3R1, PRKCD, RAG1, RAG2, RELA, RIF1, SEC61A1, SEC61A2, SEC61G, SH2D1A, SH3KBP1, STAT1, STAT3, TFRC, TNFRSF13B, TNFRSF13C, TNFRSF17, TNFRSF4, TNFSF13, TNFSF13B
7	AKT1, APCS, BCL6, BLNK, BTK, CD19, CD27, CD274, CD28, CD40, CD40LG, CD79A, CD79B, CD80, CD81, CD86, CDX1, CLEC16A, CR2, CTLA4, CXCL12, CXCR4, DCLRE1C, GATA2, ICOS, ICOSLG, IGHM, IGLL1, IKBKB, IKBKG, IL21, IL21R, IL4I1, IRF4, LRBA, LRRC32, MLH1, MS4A1, NFKB1, NFKB2, NFKBIA, P2RX7, PDCD1, PDCD1LG2, PIK3AP1, PIK3CD, PIK3R1, PRDM1, PRKCD, PRKD1, RAG1, RAG2, RELA, SEC61A1, SEC61A2, SEC61G, SH2D1A, SH3KBP1, STAT1, STAT3, TCF3, TFRC, TGFB1, TGFB2, TGFB3, TNFRSF13B, TNFRSF13C, TNFRSF17, TNFRSF18, TNFRSF4, TNFSF10, TNFSF13, TNFSF13B, VAV1, VAV2
9	ADAM17, ATG16L1, CARD9, CASP8, CASP8AP2, CD40LG, CDX1, CTLA4, CYBA, CYBB, DEFB1, DKC1, FOXP3, FUT2, GATA3, GUCY2C, ICOS, IKBKG, IKZF2, IL10, IL10RA, IL10RB, IL15, IL15RA, IL17A, IL17RA, IL1RL1, IL23A, IL23R, IL2RA, IL33, IL4I1, IRAK1, IRGM, LRBA, LRRC32, LRRK2, MYO5B, NCF1, NCF2, NCF4, NOD2, P2RX7, PLCG2, PRDM1, PTEN, RNF186, RORC, RTEL1, SH2D1A, SPNS1, STXBP2, TERC, TERT, TGFB1, TGFB2, TGFB3, TINF2, TMEM173, TTC7A, WAS, WIPF1, WRAP53, XIAP
10	ADA, ADA2, AICDA, AKT1, APCS, BCL6, BLNK, BTK, CASP8, CCR6, CD19, CD27, CD274, CD28, CD40, CD40LG, CD79A, CD79B, CD80, CD81, CD86, CDX1, CLEC16A, CR2, CTLA4, CXCL12, CXCR4, CXCR5, DCLRE1C, DOCK11, FCGRT, GATA2, HSPA5, ICOS, ICOSLG, IGHM, IGLL1, IKBKB, IKBKG, IL21, IL21R, IL4I1, IRF4, LRBA, LRRC32, LRRK2, MLH1, MS4A1, MSH2, MSH5, NBN, NFKB1, NFKB2, NFKBIA, NLRP12, P2RX7, PDCD1, PDCD1LG2, PIK3AP1, PIK3CD, PIK3R1, PRDM1, PRKCD, PRKD1, PTEN, RAD50, RAG1, RAG2, RELA, RORA, RPTOR, RTP4, SEC61A1, SEC61A2, SEC61G, SH2D1A, SH3KBP1, SOCS1, STAT1, STAT3, TCF3, TFRC, TGFB1, TGFB2, TGFB3, TMEM173, TNFAIP1, TNFRSF13B, TNFRSF13C, TNFRSF17, TNFRSF18, TNFRSF4, TNFSF10, TNFSF13, TNFSF13B, VAV1, VAV2
11	ADA, ADA2, AICDA, AKT1, APCS, BCL6, BLNK, BTK, CASP8, CCR6, CD19, CD27, CD274, CD28, CD79A, CD79B, CD80, CD81, CD86, CDX1, CLEC16A, CR2, CTLA4, CXCL12, CXCR4, CXCR5, DCLRE1C, DOCK11, FCGRT, FOXP3, GATA2, HSPA5, ICOS, ICOSLG, IGHM, IGLL1, IKBKB, IKBKG, IL21, IL21R, IL4I1, IRF4, LRBA, LRRC32, LRRK2, MLH1, MS4A1, MSH2, MSH5, NBN, NFKB1, NFKB2, NFKBIA, NLRP12, NOTCH1, NOTCH2, P2RX7, PDCD1, PDCD1LG2, PIK3AP1, PIK3CD, PIK3R1, PRDM1, PRKCD, PRKD1, PTEN, PTPN6, RAD50, RAG1, RAG2, RELA, RORA, RPTOR, RTP4, SEC61A1, SEC61A2, SEC61G, SH2D1A, SH3KBP1, SOCS1, STAT1, STAT3, TCF3, TFRC, TGFB1, TGFB2, TGFB3, TMEM173, TNFAIP1, TNFRSF13B, TNFRSF13C, TNFRSF17, TNFRSF18, TNFRSF4, TNFSF10, TNFSF13, TNFSF13B, VAV1, VAV2
14	ADA, ADA2, AICDA, AKT1, AKT3, APCS, BCL2L1, BCL6, BLNK, BTK, CASP8, CCL5, CCR6, CD19, CD27, CD274, CD28, CD79A, CD79B, CD80, CD81, CD86, CDX1, CLEC16A, CORO1B, CR2, CTLA4, CXCL12, CXCR4, CXCR5, DCLRE1C, DOCK11, DUSP2, FCGRT, FOXP3, GATA2, GRAP, GRB2, HDAC4, HSPA5, ICOS, ICOSLG, IGHM, IGLL1, IKBKB, IKBKE, IKBKG, IL21, IL21R, IL4I1, IRF4, KCNC4, KCNN4, KIDINS220, LRBA, LRRC32, LRRK2, MLH1, MS4A1, MSH2, MSH5, MTA3, NBN, NFKB1, NFKB2, NFKBIA, NLRP12, NOTCH1, NOTCH2, P2RX7, PDCD1, PDCD1LG2, PIK3AP1, PIK3CD, PIK3R1, PRDM1, PRKCD, PRKD1, PTEN, PTPN1, PTPN6, RAD50, RAG1, RAG2, RELA, RORA, RPS6KB2, RPTOR, RTP4, SEC61A1, SEC61A2, SEC61G, SH2D1A, SH3KBP1, SOCS1, STAT1, STAT3, STK11, TCF3, TFRC, TGFB1, TGFB2, TGFB3, TMEM173, TNFAIP1, TNFRSF10A, TNFRSF13B, TNFRSF13C, TNFRSF17, TNFRSF18, TNFRSF4, TNFSF10, TNFSF13, TNFSF13B, USP8, VAV1, VAV2, WNT5A, XCL1, XIAP
16	ADA, ADA2, AICDA, AKT1, AKT3, APCS, BCL2L1, BCL6, BLNK, BTK, CASP8, CCL5, CCR6, CD19, CD27, CD274, CD28, CD40, CD40LG, CD79A, CD79B, CD80, CD81, CD86, CDX1, CLEC16A, CORO1B, CR2, CTLA4, CXCL12, CXCR4, CXCR5, DCLRE1C, DOCK11, DUSP2, FCGRT, FOXP3, GATA2, GRAP, GRB2, HDAC4, HSPA5, ICOS, ICOSLG, IGHM, IGLL1, IKBKB, IKBKE, IKBKG, IKZF1, IL21, IL21R, IL4I1, IRF2BP2, IRF4, KCNC4, KCNN4, KIDINS220, LRBA, LRRC32, LRRK2, MLH1, MS4A1, MSH2, MSH5, MTA3, NBN, NFKB1, NFKB2, NFKBIA, NLRP12, NOTCH1, NOTCH2, P2RX7, PDCD1, PDCD1LG2, PIK3AP1, PIK3CD, PIK3R1, PRDM1, PRKCD, PRKD1, PTEN, PTPN1, PTPN6, RAD50, RAG1, RAG2, RELA, RORA, RPS6KB2, RPTOR, RTP4, SEC61A1, SEC61A2, SEC61G, SH2D1A, SH3KBP1, SOCS1, STAT1, STAT3, STK11, TCF3, TFRC, TGFB1, TGFB2, TGFB3, TMEM173, TNFAIP1, TNFRSF10A, TNFRSF13B, TNFRSF13C, TNFRSF17, TNFRSF18, TNFRSF4, TNFSF10, TNFSF13, TNFSF13B, USP8, VAV1, VAV2, WNT5A, XCL1, XIAP
17	ADAM17, AHR, AIRE, ATG16L1, BCL10, CARD9, CARMIL2, CASP8, CASP8AP2, CD40LG, CDX1, CLEC6A, CLEC7A, CTLA4, CYBA, CYBB, DEFB1, DKC1, DOCK8, FLG, FOXO3, FOXP3, FUT2, GATA3, GUCY2C, ICAM1, ICOS, IKBKG, IKZF2, IL10, IL10RA, IL10RB, IL12A, IL12B, IL12RB1, IL15, IL15RA, IL17A, IL17F, IL17RA, IL17RB, IL17RC, IL1RL1, IL22, IL23A, IL23R, IL25, IL2RA, IL33, IL4I1, IL6, IL6ST, IRAK1, IRAK4, IRF8, IRGM, LRBA, LRRC32, LRRK2, MRTFA, MYO5B, NCF1, NCF2, NCF4, NFAT5, NOD2, P2RX7, PGM3, PLCG2, PRDM1, PTEN, RAC2, RFXANK, RNF186, RORA, RORC, RTEL1, SH2D1A, SLC11A1, SOCS1, SPINK5, SPNS1, STAT1, STAT3, STK4, STXBP2, SYK, TERC, TERT, TGFB1, TGFB2, TGFB3, TGFBR1, TGFBR2, TINF2, TMEM173, TRAF3IP2, TTC7A, TYK2, USP8, WAS, WIPF1, WRAP53, XIAP, ZNF341
18	ADA, ADA2, AICDA, AKT1, AKT3, APCS, BCL2L1, BCL6, BLNK, BTK, CASP8, CCL5, CCR6, CD19, CD27, CD274, CD28, CD40, CD40LG, CD79A, CD79B, CD80, CD81, CD86, CDX1, CLEC16A, CORO1B, CR2, CTLA4, CXCL12, CXCR4, CXCR5, DCLRE1C, DOCK11, DUSP2, FCGRT, FOXP3, GATA2, GRAP, GRB2, HDAC4, HSPA5, ICOS, ICOSLG, IGHM, IGLL1, IKBKB, IKBKE, IKBKG, IKZF1, IL21, IL21R, IL4I1, IRF2BP2, IRF4, KCNC4, KCNN4, KIDINS220, LRBA, LRRC32, LRRK2, MLH1, MS4A1, MSH2, MSH5, MTA3, NBN, NFKB1, NFKB2, NFKBIA, NLRP12, NOTCH1, NOTCH2, P2RX7, PDCD1, PDCD1LG2, PIK3AP1, PIK3CD, PIK3R1, PRDM1, PRKCD, PRKD1, PTEN, PTPN1, PTPN6, RAD50, RAG1, RAG2, RELA, RORA, RPS6KB2, RPTOR, RTP4, SEC61A1, SEC61A2, SEC61G, SH2D1A, SH3KBP1, SOCS1, STAT1, STAT3, STAT5A, STAT5B, STK11, TCF3, TFRC, TGFB1, TGFB2, TGFB3, TMEM173, TNFAIP1, TNFRSF10A, TNFRSF13B, TNFRSF13C, TNFRSF17, TNFRSF18, TNFRSF4, TNFSF10, TNFSF13, TNFSF13B, USP8, VAV1, VAV2, WNT5A, XCL1, XIAP
19	CARMIL2, CIB1, CXCR4, DOCK2, DOCK8, GATA2, IL2, IL2RG, IL7, IL7R, RFX5, RFXANK, RFXAP, RHOH, TAP1, TAP2, TAPBP, TMC6, TMC8, WAS
20	ADA2, AICDA, AIRE, BBX, BTK, CARD11, CARD9, CARMIL2, CD274, CD28, CR2, CTLA4, DKC1, DOCK8, FCHO1, FOXP3, ICOS, IKZF1, IL10RA, IL10RB, IL17A, IL17RA, ITSN2, LRBA, MICALL2, MYH9, MYO5B, NCF2, NFKB1, NFKB2, NFKBIA, NOD2, P2RX7, PDCD1, PGM3, PIK3C2A, PIK3CD, PIK3R1, PIK3R4, PTEN, RAG1, RAG2, REL, RELA, RELB, SEC61A1, SPINK5, STAT1, STAT3, STXBP2, TNFRSF13B, TNFRSF13C, TNFRSF17, TNFSF10, TNFSF13, TNFSF13B, TYK2, WAS, ZNF341
21	ADA2, AICDA, AIRE, BBX, BTK, CARD11, CARD9, CARMIL2, CD274, CD28, CR2, CTLA4, DKC1, DOCK8, FCHO1, FOXP3, ICOS, IKZF1, IL10RA, IL10RB, IL17A, IL17RA, ITSN2, LRBA, MICALL2, MYH9, MYO5B, NCF2, NFKB1, NFKB2, NFKBIA, NOD2, P2RX7, PDCD1, PGM3, PIK3C2A, PIK3CD, PIK3R1, PIK3R4, PTEN, RAG1, RAG2, REL, RELA, RELB, SEC61A1, SH2D1A, SPINK5, STAT1, STAT3, STXBP2, TNFRSF13B, TNFRSF13C, TNFRSF17, TNFSF10, TNFSF13, TNFSF13B, TYK2, WAS, XIAP, ZNF341
27	ADA, ADA2, AICDA, AIRE, ATM, BACH2, BANK1, BCL11B, BCL2L11, BLK, BLNK, BTK, CAMLG, CARD11, CARD9, CD19, CD40, CD40LG, CD79A, CD79B, CD81, CFTR, CLEC7A, CR2, CTLA4, CXCR4, DCLRE1C, DKC1, DOCK8, EBF1, FANCA, FANCE, FASLG, FCGR3B, GATA2, ICOS, IFIH1, IGHM, IKBKB, IKBKG, IKZF1, IKZF3, IL12A, IL12RB1, IL17A, IL17F, IL17RA, IL17RC, IL21, IL21R, IL23A, IL23R, IL2RG, IL6, INPP5D, IRAK4, IRF2BP2, IRF3, IRF8, LRBA, MAGT1, MBL2, MCM4, MDC1, MRE11, MS4A1, MYC, NEIL1, NFKB1, NFKB2, NFKBIA, NHEJ1, NLRC4, NLRP12, NLRP2, NLRP3, ORAI1, PGM3, PIK3CD, PIK3R1, PLCG2, PMS2, POLE, PRKCD, PRKDC, PRRC2A, PTPN22, PTPRC, RAC2, RAG1, RAG2, RNASEH2A, RNASEH2B, RNASEH2C, RTEL1, SAMD9L, SAMHD1, SATB1, SEC61A1, SEC61A2, SEC61B, SEC61G, SH2D1A, SH3BP2, SKIV2L, SPINK5, STAT1, STAT3, STIM1, STXBP2, TAP2, TCF3, TINF2, TLR3, TNFAIP3, TNFRSF11A, TNFRSF13B, TNFRSF13C, TNFSF12, TNFSF13, TNFSF13B, TNFSF15, TNIP1, TRAF3IP2, TRNT1, TWIST1, TYK2, UNG, VAV1, WAS, XIAP, ZNF341
29	ADA, ADA2, AICDA, AIRE, ATM, BACH2, BANK1, BCL11B, BCL2L11, BLK, BLNK, BTK, CAMLG, CARD11, CARD9, CD19, CD40, CD40LG, CD79A, CD79B, CD81, CFTR, CLEC7A, CR2, CTLA4, CXCR4, DCLRE1C, DKC1, DOCK8, EBF1, FANCA, FANCE, FASLG, FCGR3B, GATA2, ICOS, IFIH1, IGHM, IKBKB, IKBKG, IKZF1, IKZF3, IL12A, IL12RB1, IL17A, IL17F, IL17RA, IL17RC, IL21, IL21R, IL23A, IL23R, IL2RG, IL6, INPP5D, IRAK4, IRF2BP2, IRF3, IRF8, LRBA, MAGT1, MBL2, MCM4, MDC1, MRE11, MS4A1, MYC, NEIL1, NFKB1, NFKB2, NFKBIA, NHEJ1, NLRC4, NLRP12, NLRP2, NLRP3, ORAI1, PGM3, PIK3CD, PIK3R1, PLCG2, PMS2, POLE, PRKCD, PRKDC, PRRC2A, PTPN22, PTPRC, RAC2, RAG1, RAG2, RNASEH2A, RNASEH2B, RNASEH2C, RTEL1, SAMD9L, SAMHD1, SATB1, SEC61A1, SEC61A2, SEC61B, SEC61G, SH2D1A, SH3BP2, SKIV2L, SPINK5, STAT1, STAT3, STIM1, STXBP2, TAP2, TCF3, TINF2, TLR3, TNFAIP3, TNFRSF11A, TNFRSF13B, TNFRSF13C, TNFSF12, TNFSF13, TNFSF13B, TNFSF15, TNIP1, TRAF3IP2, TRNT1, TWIST1, TYK2, UNG, VAV1, WAS, XIAP, ZNF341
30	ADA, ADA2, AICDA, AIRE, ATM, BACH2, BANK1, BCL11B, BCL2L11, BLK, BLNK, BTK, CAMLG, CARD11, CARD9, CD19, CD40, CD40LG, CD79A, CD79B, CD81, CFTR, CLEC7A, CR2, CTLA4, CXCR4, CYBA, CYBB, DCLRE1C, DKC1, DOCK8, EBF1, FANCA, FANCE, FASLG, FCGR3B, FOXP3, GATA2, ICOS, IFIH1, IGHM, IKBKB, IKBKG, IKZF1, IKZF3, IL12A, IL12RB1, IL17A, IL17F, IL17RA, IL17RC, IL21, IL21R, IL23A, IL23R, IL2RG, IL6, INPP5D, IRAK4, IRF2BP2, IRF3, IRF8, LRBA, MAGT1, MBL2, MCM4, MDC1, MRE11, MS4A1, MYC, NCF1, NCF2, NCF4, NEIL1, NFKB1, NFKB2, NFKBIA, NHEJ1, NLRC4, NLRP12, NLRP2, NLRP3, ORAI1, PGM3, PIK3CD, PIK3R1, PLCG2, PMS2, POLE, PRKCD, PRKDC, PRRC2A, PTPN22, PTPRC, RAC2, RAG1, RAG2, RNASEH2A, RNASEH2B, RNASEH2C, RTEL1, SAMD9L, SAMHD1, SATB1, SEC61A1, SEC61A2, SEC61B, SEC61G, SH2D1A, SH3BP2, SKIV2L, SPINK5, STAT1, STAT3, STIM1, STXBP2, TAP2, TCF3, TINF2, TLR3, TNFAIP3, TNFRSF11A, TNFRSF13B, TNFRSF13C, TNFSF12, TNFSF13, TNFSF13B, TNFSF15, TNIP1, TRAF3IP2, TRNT1, TWIST1, TYK2, UNG, VAV1, WAS, XIAP, ZNF341
33	ADA, ADA2, AICDA, AIRE, ATM, BACH2, BANK1, BCL11B, BCL2L11, BLK, BLNK, BTK, CAMLG, CARD11, CARD9, CD19, CD40, CD40LG, CD79A, CD79B, CD81, CFTR, CLEC7A, CR2, CTLA4, CXCR4, CYBA, CYBB, DCLRE1C, DKC1, DOCK8, EBF1, FANCA, FANCE, FASLG, FCGR3B, FOXP3, GATA2, ICOS, IFIH1, IGHM, IKBKB, IKBKG, IKZF1, IKZF3, IL12A, IL12RB1, IL17A, IL17F, IL17RA, IL17RC, IL21, IL21R, IL23A, IL23R, IL2RG, IL6, INPP5D, IRAK4, IRF2BP2, IRF3, IRF8, LRBA, MAGT1, MBL2, MCM4, MDC1, MRE11, MS4A1, MYC, MYO9A, NCF1, NCF2, NCF4, NEIL1, NFKB1, NFKB2, NFKBIA, NHEJ1, NLRC4, NLRP12, NLRP2, NLRP3, ORAI1, PGM3, PIK3CD, PIK3R1, PIK3R4, PLCG2, PMS2, POLE, PRKCD, PRKDC, PRRC2A, PTPN22, PTPRC, RAC2, RAG1, RAG2, RNASEH2A, RNASEH2B, RNASEH2C, RTEL1, SAMD9L, SAMHD1, SATB1, SEC61A1, SEC61A2, SEC61B, SEC61G, SH2D1A, SH3BP2, SKIV2L, SPINK5, STAT1, STAT3, STIM1, STXBP2, TAP2, TCF3, TINF2, TLR3, TNFAIP3, TNFRSF11A, TNFRSF13B, TNFRSF13C, TNFSF12, TNFSF13, TNFSF13B, TNFSF15, TNIP1, TRAF3IP2, TRNT1, TWIST1, TYK2, UNG, VAV1, WAS, XIAP, ZNF341

Supplementary Table 2. Short lists of candidate variants were generated from our internal database based on an (individual) frequency below 1% in our internal cohort or in the Genome Aggregation Database (gnomAD) - exomes and genomes - cohort, and a "high" or "moderate" predicted impact.

Type of variant	Predicted impact	Sequence Ontology ID
frameshift_variant	HIGH	SO:0001589
rare_amino_acid_variant	HIGH	SO:0002008
stop_gained	HIGH	SO:0001587
chromosome_number_variation	HIGH	SO:1000182
transcript_ablation	HIGH	SO:0001893
exon_loss_variant	HIGH	SO:0001572
frameshift_elongation	HIGH	SO:0001909
frameshift_truncation	HIGH	SO:0001910
internal_feature_elongation	HIGH	SO:0001908
feature_truncation	HIGH	SO:0001906
mnv	HIGH	SO:0002007
complex_substitution	HIGH	SO:1000005
stop_lost	HIGH	SO:0001578
start_lost	HIGH	SO:0002012
splice_acceptor_variant	HIGH	SO:0001574
splice_donor_variant	HIGH	SO:0001575
missense_variant	MODERATE	SO:0001583
inframe_insertion	MODERATE	SO:0001821
disruptive_inframe_insertion	MODERATE	SO:0001824
inframe_deletion	MODERATE	SO:0001822
disruptive_inframe_deletion	MODERATE	SO:0001826
5_prime_utr_truncation	MODERATE	SO:0002013
3_prime_utr_truncation	MODERATE	SO:0002015
splice_region_variant	MODERATE	SO:0001630
stop_retained_variant	LOW	SO:0001567
initiator_codon_variant	LOW	SO:0001582
synonymous_variant	LOW	SO:0001819
coding_transcript_intron_variant	LOW	SO:0001969
non_coding_transcript_exon_variant	LOW	SO:0001792
non_coding_transcript_intron_variant	LOW	SO:0001970
5_prime_UTR_premature_start_codon	LOW	SO:0001988
5_prime_utr_variant	LOW	SO:0001623
3_prime_utr_variant	LOW	SO:0001624
direct_tandem_duplication	MODIFIER	SO:1000039
upstream_gene_variant	MODIFIER	SO:0001631
downstream_gene_variant	MODIFIER	SO:0001632
intergenic_variant	MODIFIER	SO:0001628
tf_binding_site_variant	MODIFIER	SO:0001782
regulatory_region_variant	MODIFIER	SO:0001566
conserved_intron_variant	MODIFIER	SO:0002018
intragenic_variant	MODIFIER	SO:0002011
conserved_intergenic_variant	MODIFIER	SO:0002017
structural_variant	MODIFIER	SO:0001537
coding_sequence_variant	MODIFIER	SO:0001580
intron_variant	MODIFIER	SO:0001627
exon_variant	MODIFIER	SO:0001791
splicing_variant	MODIFIER	SO:0001568
miRNA	MODIFIER	SO:0000276
gene_variant	MODIFIER	SO:0001564
coding_transcript_variant	MODIFIER	SO:0001968
non_coding_transcript_variant	MODIFIER	SO:0001619
transcript_variant	MODIFIER	SO:0001576
intergenic_region	MODIFIER	SO:0000605
chromosome	MODIFIER	SO:0000340
sequence_variant	MODIFIER	SO:0001060
mature_miRNA_variant	MODIFIER	SO:0001620
protein_altering_variant	MODERATE	SO:0001818
incomplete_terminal_codon_variant	LOW	SO:0001626

Supplementary Figure 1. Quality control data: Each dot represents one sample, which was sequenced in one of the 45 individual runs using one of the 18 unique gene panel designs based on HaloPlex (left) and SureSelect (right) enrichment technology. (A) The mean sample read depth per run. (B) The mean coverage of samples per sequencing run. Colours indicate different panel designs.

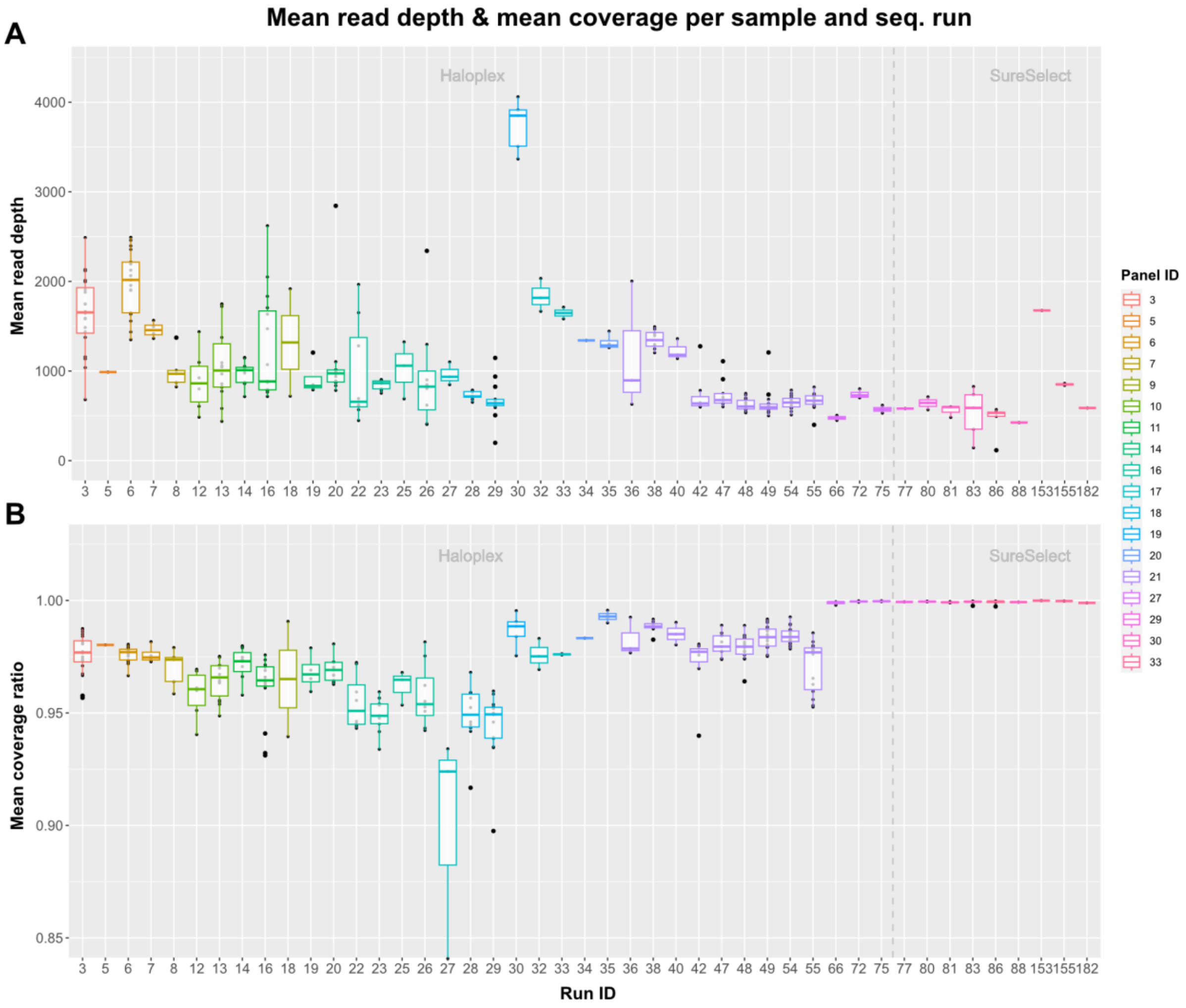

Supplementary Figure 2. **The number of detected variants correlates with increasing number of genes screened per panel.** Each dot represents the number of variants detected per sample (Y-axis) vs the number of base pairs sequenced (X-axis), which correlates with the number of genes screened per sample (colour scale). Since 18 different panel designs were used and a few samples were sequenced in more than one run, the number of genes screened per sample varies across the cohort. (A) Total variants per subject. (B) Number of rare variants per subject, filtered only by allele frequency. (C) Rare variants with "High" or "Moderate" predicted impact. (D) Total number of genes sequenced per subject for the 291 individuals.

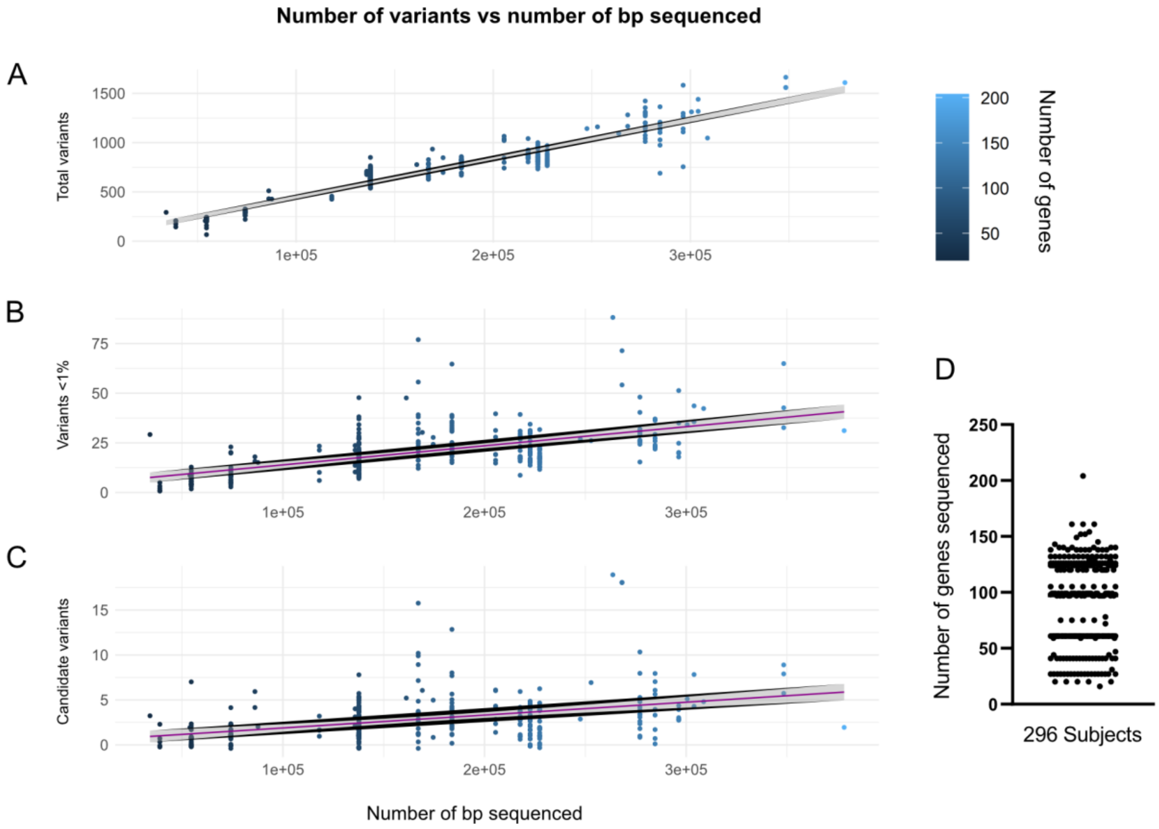

Supplementary Figure 3. A) Age distribution at first clinical manifestation. B) Distribution of molecularly diagnosed individuals per mutated gene, age at diagnosis and gender.

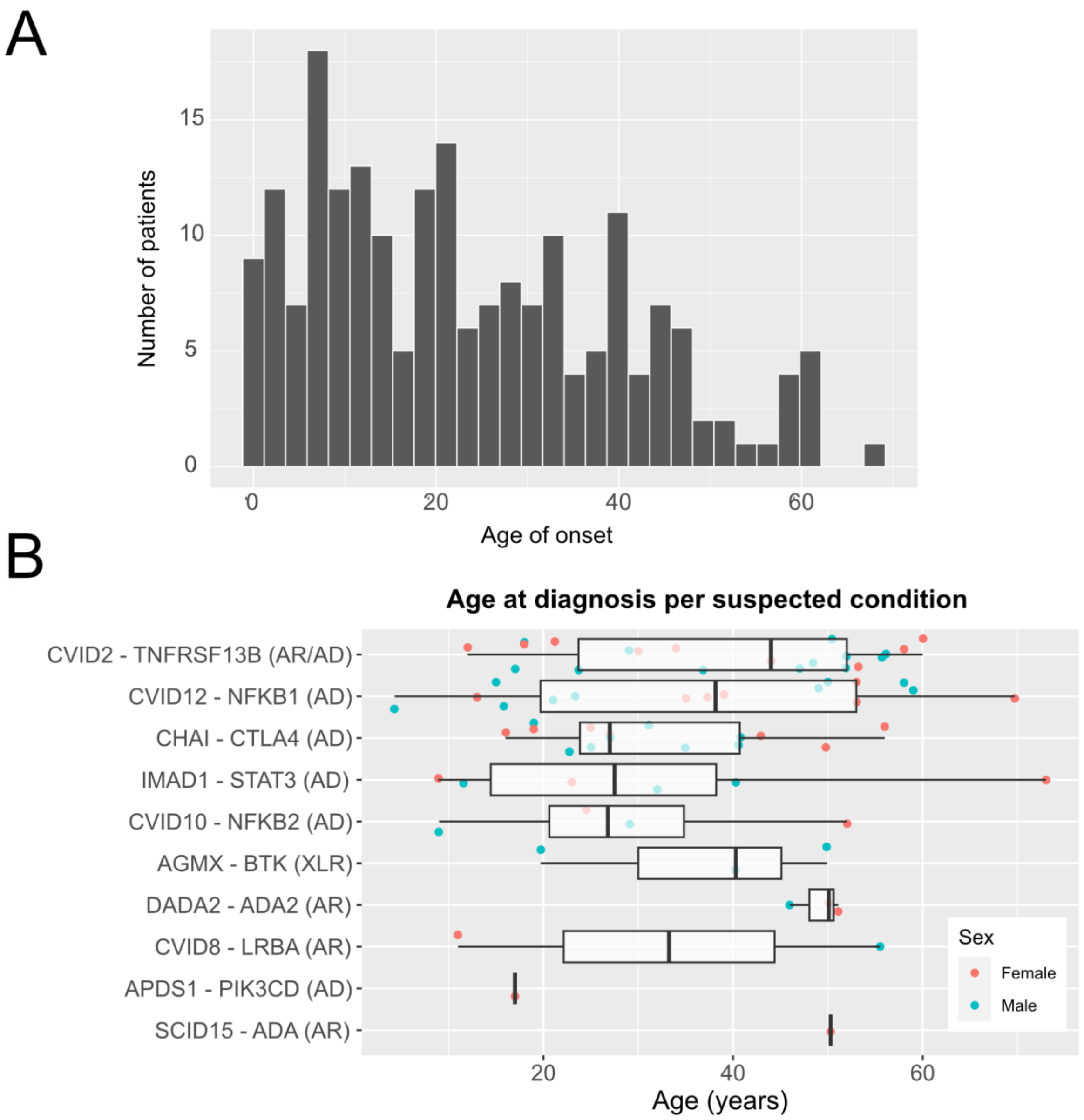

Supplementary Figure 4. **Functional assessment of a newly identified *BTK* variant.** (A) Normal BTK expression in CD19+ B cells but slightly reduced in monocytes from patient compared to a healthy donor. (B) Ca^{+2} mobilization kinetics by means of Indo-1 is attenuated in naïve CD19 B cells derived from the BTK-mutated patient (red) in contrast to three healthy donors (light grey, dark grey and black). Anti-IgM stimulation and addition of Ionomycin is indicated by arrows. (C) Histogram overlays show equivalent phosphorylation of Ig□, SLP65 and BTK in naïve $CD19^{+}CD21^{+}$ B cells of patient (red) compared to a healthy donor (blue). Unstimulated (solid lines) grey and black in a healthy donor and patient samples respectively.

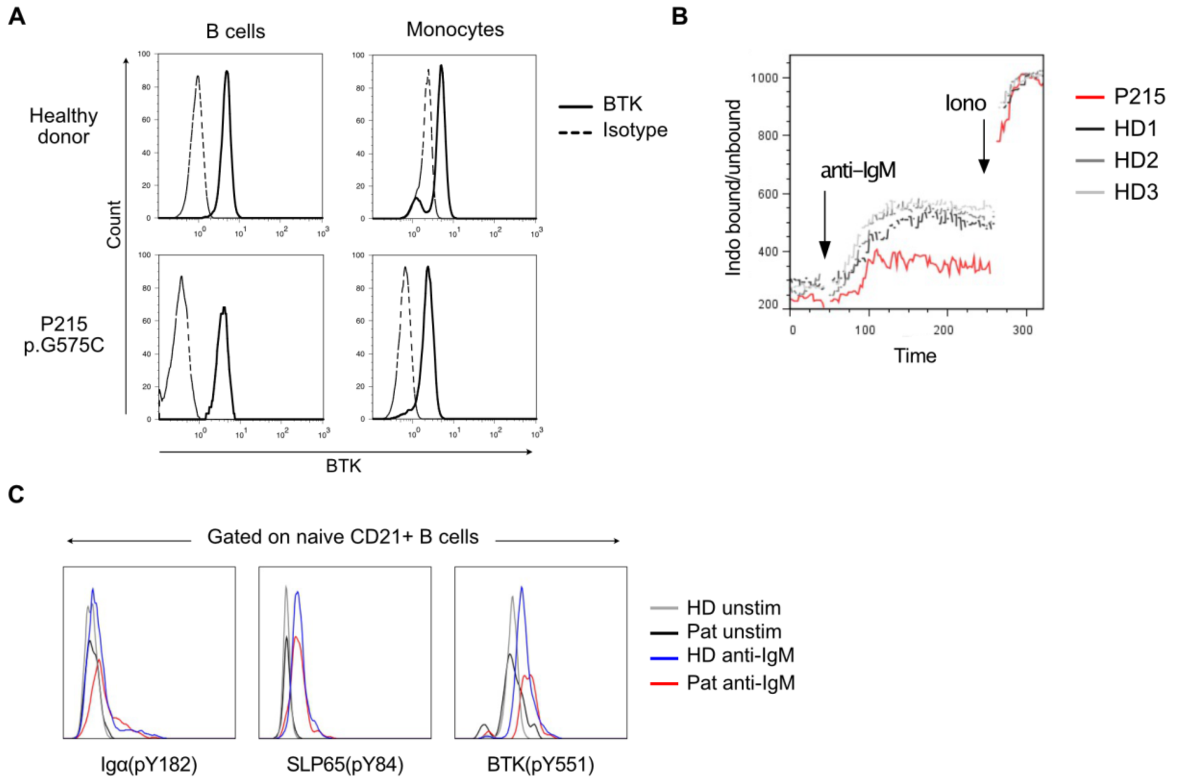

Supplementary Table 3. Summary of different TGP or targeted WES sequencing studies in patients with PID.

Study	Year	Syndrome	*N* of patients analyzed	Consanguinity (%)	Sequencing technique	*N* of genes analyzed	Coverage (%)	Reading depth	Molecular Diagnosis (%)	*N* of variants identified	*N* of new variants identified	Ref (PMID)
This study	2021	PAD	296	Non-consanguinity	TGP	27 to 286 genes	>90 (HaloPlex) >98 (SureSelect)	300X to 4200X (HaloPlex) 50X to 1700X (SureSelect)	77 (25.68%)	64	17	-
Fusaro *et al.*	2020	PID	129	16	TGP	300	99	539X ± 203X	40 (31%)	46	26	32531373
Abolhassani *et al.*	2020	CVID	571	63 within the Iranian group	WES	344	na	na	232 (40.6%)	na	na	31942606
Rudilla *et al.*	2019	PID	61	23	CES	260	89 ± 4	81X ± 28X	19 (31%)	22	12	31681265
Arts *et al.*	2019	PID	254	na	WES	302	95.3	120.7X to 130.2X	72 (28%)	na	na	31203817
Abolhassani *et al.*	2019	PAD	126	82.5	WES	378	na	na	86 (68.2%)	66	na	29921932
Chi *et al.*	2018	PID	56	na	TGP	171	99.9	na	13 (23.2%)	117	12	30290665
Abolhassani *et al.*	2018	CID	243	76	WES TGP	365 200	50X 335X	na	189 (77.8%)	na	na	28916186
Bisgin *et al.*	2018	PID	37	92	TGP	60	na	200X	17 (46%)	17	5	29888287
Rae *et al.*	2018	PID	27	na	TGP	242	96.2 to 99.5	98X	13 (48%)	15	8	29077208
Stray-Pedersen *et al.*	2016	PID	278	6	WES	475	>90	>100X	110 (40%)	148	10	27577878
Maffucci *et al.*	2016	CVID	50	Non-consanguinity	WES	269	na	na	15 (30%)	17	13	27379089
Gallo *et al.*	2016	PID	45	na	TGP<br><br>WES	571<br><br>-	98.9<br><br>97	580X<br><br>>10X	3 (11%)<br><br>4 (22%)	8	na	27872624
Al-Mousa *et al.*	2016	PID	139	90	TGP	162	96.5	461X	35 (25%)	23	na	26915675
Kojima *et al.*	2016	PID	59	na	TGP	349	99.1	na	8 (14%)	9	na	26997321
Moens *et al.*	2014	PID	15	20	TGP	179	88	1304X±662	6 (47%)	7	na	25502423
Stoddard *et al.*	2014	PID	120	na	TGP	173	99.53	305X	18 (15%)	na	na	25404929
Nijman *et al.*	2014	PID	26	4	TGP	170	90.4	338X (array enrichment) 192X (SureSelect)	4 (15%)	5	2	24139496

PID: Primary immunodeficiencies, CVID: common variable immunodeficiency, SCID: severe combined immunodeficiency, HLH: hemophagocytic lymphohistiocytosis, PAD: Predominantly antibody deficiency, TGP: Targeted gene panel, WES: Whole exome sequencing, CES: Clinical exome sequencing N: number, na: not available.
